# Impact of digital health on Type 2 diabetes management: a randomised controlled trial of the ‘TreC Diabete’ platform (TELEMECHRON Study)

**DOI:** 10.3389/fcdhc.2025.1589548

**Published:** 2025-06-10

**Authors:** Alexia Giovanazzi, Lorenzo Gios, Maria Adalgisa Gentilini, Marina Mastellaro, Patrizia Bartolotta, Lucrezia Nicolussi Giacomaz, Claudio Eccher, Sandro Inchiostro

**Affiliations:** ^1^ Servizio Governance Clinica, Azienda Provinciale per i Servizi Sanitari della Provincia Autonoma di Trento, Trento, Italy; ^2^ TrentinoSalute4.0, Competence Center for Digital Health, Trento, Provincia Autonoma di Trento, Italy; ^3^ Unità Operativa epidemiologica clinica e valutativa, Azienda Provinciale per i Servizi Sanitari della Provincia Autonoma di Trento, Trento, Provincia Autonoma di Trento, Italy; ^4^ Digital Health Innovation Lab Unit, Fondazione Bruno Kessler, Trento, Provincia Autonoma di Trento, Italy; ^5^ Dipartimento medico, Azienda Provinciale per i Servizi Sanitari della Provincia Autonoma di Trento, Trento, Italy

**Keywords:** type 2 diabetes, metabolic control, randomised controlled trial, mHealth, telemedicine, digital health

## Abstract

**Background:**

The use of technologies in the health field has progressively increased. Within the context of a broader project funded by the Italian Ministry of Health (TELEMECHRON study), a randomised controlled trial (RCT) has been conducted in the Autonomous Province of Trento on type 2 diabetes individuals with an untargeted glycated haemoglobin (HbA1c) level.

**Methods:**

The overall aim was to evaluate the impact of the “TreC Diabete” digital platform, including a smartphone application (app) and a dashboard. This open-label, parallel-group, 1:1 allocation ratio RCT in which the intervention group used the app for data entry, symptoms questionnaire, communication with healthcare staff and medication recording, while the control group received standard care. The primary endpoint was change in HbA1c levels at 12 months between groups.

**Results:**

Between December 2022 and August 2023, 103 participants were enrolled (51 intervention; 52 control), with a median age of 67 years old, time from diabetes diagnosis to enrolment 13 years, and 72% male. At 12 months, the median change in HbA1c levels did not differ significantly between groups. Regarding app usage, data entries decreased significantly from the first quarter to the second quarter but subsequently stabilised (p = 0.001). System usability (from 42 responders in the intervention group) had a median score of 95 (range: 0–100), indicating a high level of satisfaction with the platform.

**Discussion:**

The study faced several challenges, including platform technical issues, service interruption, data entry anomalies and difficulties in participant recruitment. Study generalisability may be limited by the sample’s demographics, as the trial predominantly included younger male individuals with a specific HbA1c level.

**Conclusion:**

The study highlighted key factors for future implementations, including understanding technology benefits, addressing adoption barriers, and providing education and support to both patients and healthcare providers.

## 1 Introduction

### 1.1 Background and rationale

Diabetes mellitus, particularly type 2 diabetes (T2DM), represents a major global public health burden, accounting for approximately 90% of all diabetes cases (1). As of recent estimates, over 422 million people worldwide are living with diabetes, with an annual mortality of approximately 1.5 million deaths (1–4). The chronic nature of this condition, along with its microvascular and macrovascular complications, leads to progressive damage across multiple organ systems, significantly reducing patients’ quality of life and imposing a heavy economic strain on healthcare systems and society at large (1–4).

Given the increasing prevalence of diabetes and the complex needs of affected patients, the development of innovative and sustainable healthcare models is an urgent priority. In particular, the integration of digital health tools and telemedicine within chronic disease management frameworks has emerged as a promising strategy to enhance healthcare delivery, improve self-management, and reduce avoidable hospitalisations (5). In this context, mobile health (mHealth) solutions - defined as medical and public health practices supported by mobile devices - are gaining increasing attention for their potential to improve diabetes care (6). A growing body of evidence suggests that mHealth interventions can enhance patient engagement, promote treatment adherence, and facilitate timely communication between patients and healthcare providers, potentially leading to better glycaemic outcomes. However, the clinical effectiveness of mHealth tools, including those delivered via telemedicine platforms, remains inconsistent, with some studies showing limited or variable impact on metabolic, and in the large majority of cases studies were assessing a single-function application. In these cases, telemedicine supported by mHealth seemed to be more frequently associated with improved glycaemic control, whilst past evidence did not consistently demonstrate a clear benefit of telemedicine on metabolic control (7–9).

In the Autonomous Province of Trento (PAT), approximately 29,000 individuals are affected by T2DM, representing 5.2% of the population (10). In the ‘Telemedicine for the home management of patients with chronic diseases and comorbidities analysis of current models and design of innovative strategies to improve the quality of care and optimise the use of resources: TELEMECHRON study (11), an Italian Ministry of Health co-funded project on telemedicine and its application in managing chronic diseases, PAT participants with T2DM were offered to use an application called “TreC Diabete” as part of their care plan. This application is part of a mHealth platform conceived to strengthen the continuity of care by enhancing communication between patients, caregivers, and multidisciplinary healthcare teams. Its features include chats, a digital diary for self-monitoring, secure messaging with healthcare professionals, medication reminders, and personalised feedback. Through remote monitoring and feedback, the platform aims to foster a sense of support, promote active patient participation, and streamline clinical decision-making.

Although the study was initially conceived in 2018 - within the Telemechron large study and when large-scale evidence on telemedicine in diabetes was still emerging - this study remains highly relevant. In contrast to many earlier interventions focused on single digital functions (12), “TreC Diabete” was tested as a comprehensive, multifunctional platform embedded within a real-world care pathway. The trial uniquely aimed to evaluate not only clinical outcomes but also patient-reported benefits. This makes it one of the few examples where a fully integrated digital tool—with synchronous and asynchronous features—was assessed through an RCT as a complement to standard care for T2DM. The study also offers broader insights into how mHealth platforms can be sustainably embedded into everyday clinical practice to meet the complex needs of people living with chronic diseases.

### 1.2 Objectives

#### 1.2.1 Primary objective

Evaluate the impact of a new care model supported using a digital platform “TreC Diabete” on glycated haemoglobin (HbA1c) levels change over a 12-month period.

#### 1.2.2 Secondary objective

Evaluate the impact of using the platform in terms of blood pressure (BP) and lipid control, body weight change, physical activity, hospitalisations, specialist visits, prescriptions adherence level, quality of life, and usability of the “TreC Diabete” app at different time points.

## 2 Methods

### 2.1 Trial design

Randomized, controlled, superiority, with two parallel groups and 1:1 ratio.

### 2.2 Methodological changes

An amendment was submitted to remove two eligibility criteria due to a small number of eligible subjects with initially planned characteristics (about 20 participants in June and July 2022).

### 2.3 Participants

Individuals who were offered participation had a scheduled visit to one of the diabetes centres of the province. Participants had to be 18 to 85 years old, with an HbA1c between 53 mmol/mol (7%) and 108 mmol/mol (12%), able to walk without aids, have access to a smartphone (personally or through a caregiver), and consent to use the app for entering data.

Exclusion criteria included individuals with a body mass index (BMI) below 18 kg/m² or above 45 kg/m², systolic blood pressure (sBP) under 100 mmHg and/or diastolic blood pressure (dBP) under 50 mmHg, or over 200 mmHg and/or 120 mmHg, chronic kidney failure (CKD – glomerular filtration rate eGFR <15 ml/min/m² using the CKD-Epidemiology Collaboration equation) (13), or heart failure in NYHA class IV (14). Additionally, individuals who were poorly compliant, unable or unwilling to use mobile technology, or had conditions such as life expectancy under one year or ongoing neoplastic condition were excluded.

### 2.4 Settings

Outpatient diabetes centres of the two main provincial hospitals. They are responsible for diagnosing and treating diabetes, as well as preventing its complications.

### 2.5 Interventions

Once consent to participate was obtained, study subjects were randomised into the intervention or control group. For all participants, the treatment goals were identified following the: targets and values according to national and international guidelines (15–17):

- Glycaemic target: HbA1c < 53 mmol/mol
- Blood pressure targets: sPA < 130 mmHg if < 65 years and < 140 mmHg if ≥ 65 years; dPA ≥ 70 mmHg and < 80 mmHg
- Lipid target: LDL Cholesterol < 70 mg/dl; Non-HDL Cholesterol < 100 mg/dl (if triglycerides ≥ 200 mg/dl)

Intervention group: Fifty-one individuals were assigned to this group and prescribed the “TreC Diabete”, part of the “TreC Diabete” platform developed by the Bruno Kessler Foundation (FBK). The platform includes a smartphone/tablet app for participants and a clinical dashboard for healthcare professionals.

Participants were supported by the research team in installing the app, which involved accepting an information form and entering their social security number and a 15-minute unique activation code provided by a clinician. After installation, participants were prescribed the app for 12 months with a personalised care plan, requiring them to enter clinical parameters (e.g. BP, blood glucose, weight, or heart rate - HR) and medication information with a minimum frequency of once a week The app allowed participants to exchange images and multimedia files with healthcare staff (e.g. test results or glycaemic diaries photos).

**Figure 1:**
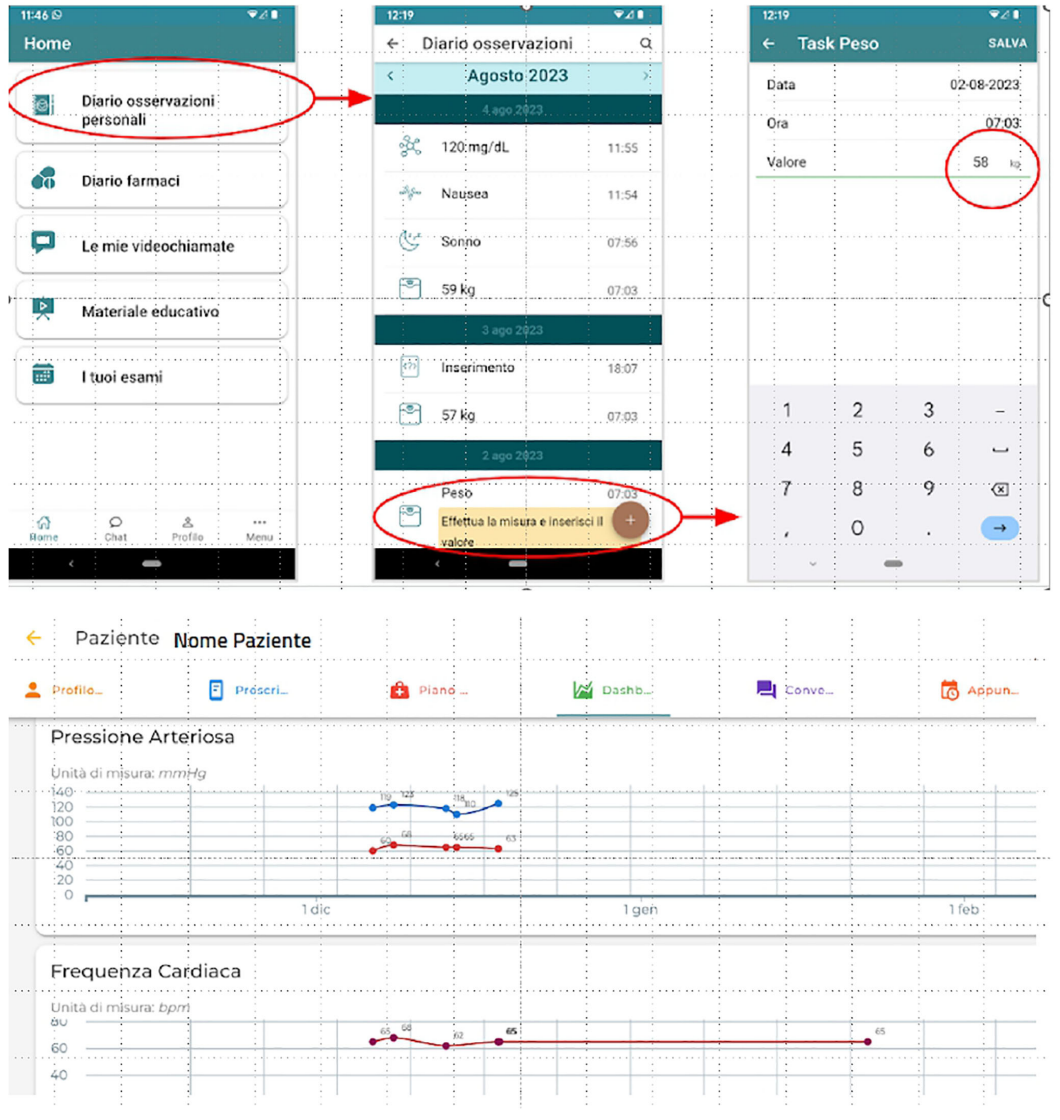
**“**TreC Diabete” app user interface and dashboard interface.

During the study, clinicians reviewed participant data at least every 45 days and monitored the subject-healthcare staff chat to address questions, provide feedback, and encourage follow-up appointments. Examples of the app user and the dashboard interface of the “TreC Diabete” platform are provided in **Figure 1**. As per Preventive Diagnostic Therapeutic Care Pathways (PPDTA) of APSS, this group maintained scheduled televisits at 6 months, unless earlier intervention was needed.

Control group: Participants adhered to the PPDTA of APSS, following the standard care for individuals with diabetes. This included a scheduled televisit at 6 months and an in-person visit at 12 months, unless they required earlier intervention. This group was instructed to record health data using their preferred method, such as a paper diary.

### 2.6 Primary outcome

Change in HbA1c levels at 12 months in the intervention group compared with the control group. Blood samples were collected by trained nurses and processed by provincial testing laboratories.

### 2.7 Secondary outcomes

These included: HbA1c < 53 mmol/mol at 12 months in both groups; BP and lipid profile targets at 12 months; average HbA1c, body weight, BP at 3, 6, and 9 months; hypoglycaemia episodes at 12 months; physical activity change at 3, 6, 9, and 12 months; medication adherence change at 12 months; system satisfaction and usability in the intervention group at 12 months; and quality of life at 12 months. Additionally, telemedicine visits and chat interactions (patient-nurse, patient-clinician), (tele)specialist visits and medication changes at 3, 6, 9, 12 months and time spent on telemedicine visits at 6 months.

Additionally, as an exploratory *post-hoc* analysis, not specified in the protocol, we investigated three distinct domains: A) the number of successful therapy intake entries; B) the number of measurements recorded by participants, based on their personalised prescription; C) the number of conversations initiated by participants or by the healthcare staff.

### 2.8 Data collection

Data were collected through an electronic-Case Report Form (eCRF) through several approaches:

- Interview: structured in-person interviews at baseline and 12 months, and structured telephone interviews to collect clinical data, hypoglycaemia episodes, and information on weight, BP checks, and physical activity during intermediate follow-up.
- Questionnaires, of which the interpretation and the scoring method are better described in the protocol (6):
  1. Quality of Life Questionnaire - Short Form - SF-12: assessing physical health (SF-PCS-12) and mental health (SF-MCS-12) domains (18) (19).
  2. Duke Activity Status Index - DASI scale: measuring participant’s perceived functional capacity (20).
  3. Physical Activity Questionnaire Daily – IPAQ: tracking physical activity over the past week (21).
  4. Morisky Medication Adherence Scale in the 8-item version (MMAS-8 Scale)^1^: evaluating medication adherence (22–25).
  5. System Usability Scale (SUS): assessing app usability and user satisfaction (26).
- Consultation of HIS or other databases for blood test, instrumental tests, specialist visits, anamnestic information, medication changes, hospitalisations.

### 2.9 Outcomes variation

Outcomes that were not evaluated or evaluated differently than initially planned, included:

- Time spent on telemedicine visits was set by clinician’s schedules at the centres and could not be adjusted for the study.
- Nurse-related outcomes as nurses at the two centres did not actively participate.
- In the intervention group, participants did not regularly provide data using the app, likely due to non-adherence to the expected use of the app). Consequently, data intended to be collected as part of the outcomes was not systematically gathered through the app. Researchers decided to collect data via phone calls instead and to apply this data collection method to the control group as well.
- Blood tests were not always performed on the required dates, mainly due to participants’ personal reasons.
- Scheduled visits, outside the study’s scope, were not always cancelled and often overlapped with study visits, making it difficult to track the chosen visit method (i.e., telemedicine)

Given the availability of data on app usage, a dedicated section was added to report adherence to app use in the intervention group.

### 2.10 Sample size

We assumed that at the end of follow-up the mean HbA1c in patients is 49 ± 3 mmol/mol in the intervention group and 52 ± 7 mmol/mol in the control group and to achieve a statistical power of 80%, accepting an alpha error of 5% and a drop-out of 20%, a sample size of 120 people was planned. The sample size was calculated using OpenEpi (free software, https://www.openepi.com/Menu/OE_Menu.htm).

### 2.11 Randomisation and allocation

Block randomization of 10 after stratification by centre was used to assign participants to one of the groups. The list was generated using the statistical program SAS software version 9.4 (SAS Institute Inc.). The list was managed by a statistician from the Clinical and Evaluation Epidemiology Unit of APSS not involved in the enrolment of participants. Opaque envelopes, each marked with an alphanumeric code (increasing numbers) were used to conceal information regarding the assignment to one of groups. Opaque envelopes were opened sequentially by a team member not aware of group assignment. Once the envelopes were opened, the participant was immediately informed about the allocated group.

### 2.12 Blindness

The study was conducted in an open-label setting: once the envelopes were opened, participants, clinicians and the data collector were aware of the allocation. Data analysis was performed by evaluators using pseudonymised data.

### 2.13 Statistical methods

The primary analysis followed the intention-to-treat (ITT) principle, including all patients in their originally assigned groups regardless of adherence. Missing data were handled using multiple imputation by chained equations (MICE) when feasible (≤ 5% missing, completely at random).

Sensitivity analyses assessed robustness using three scenarios:

- Intervention group missing data imputed with the most extreme negative values, control group with the most extreme positive values.
- Control group missing data imputed with the most extreme negative values, intervention group with the most extreme positive values.
- No imputation applied.

These analyses were also conducted using per-protocol (PP) and as-treated (AT) populations. PP analyses excluded patients with major protocol deviations (e.g., lost to follow-up, intervention group participants with < 6 monthly app entries). AT analyses categorized patients by the treatment they received. App engagement was arbitrarily defined as having ≥ 6 recorded entries per month, to distinguish active users from noncompliant participants.

Descriptive statistics summarized sociodemographic, clinical, and questionnaire data. Continuous variables were reported as medians, and interquartile ranges, while categorical variables were expressed as counts and percentages. Since most variables were non-normally distributed (Kolmogorov-Smirnov test), nonparametric methods were used: Wilcoxon rank sum test (independent samples), Wilcoxon signed-rank test (dependent samples), Friedman test (repeated measures), chi-square, and Fisher’s exact test. A two-sided significance level of 0.05 was applied.

Analyses were performed using SAS software version 9.4 and R 4.3.3 (27).

## 3 Results

Between December 2022 and August 2023, 104 participants were initially enrolled; however, the final number of participants included in the study was 103, with 51 in the intervention group and 52 in the control group. One participant in the intervention group was excluded after randomisation for meeting an exclusion criterion (BMI greater than 45 kg/m²).

**Figure 2:**
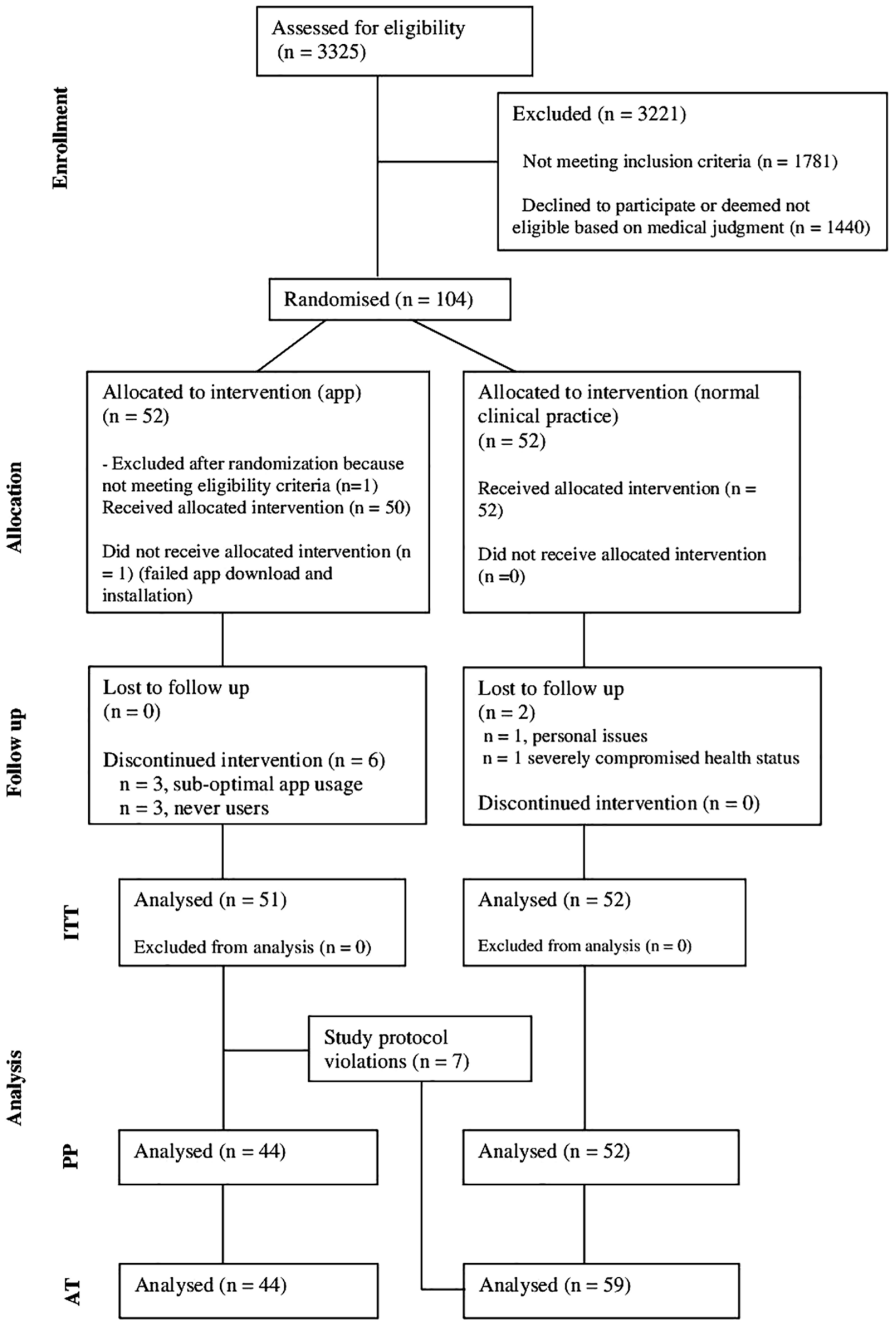
Adapted Consolidated Standard of Reporting Trials flow chart – from eligibility assessment to analysis (28).

**Figure 2** shows an adapted version of the Consolidated Standard of Reporting Trials (CONSORT) participant flowchart (28).

### 3.1 Baseline characteristics

Demographic, clinical, medical history, biohumoral, and medication characteristics at baseline are detailed in **Table 1**. These data indicate that the two randomised groups were well or sufficiently balanced, with comparable characteristics.

**Table 1:** Baseline demographics, clinical, anamnestic, biohumoral characteristics, and questionnaires scores, by assignment group and the total group.

Variable	Intervention N = 51^1^	Control N = 52^1^	Total N = 103^1^
Sex			
* Female*	18 (35%)	11 (21%)	29 (28%)
* Male*	33 (65%)	41 (79%)	74 (72%)
Age (years)	66 (61, 70)	68 (59, 72)	67 (59, 72)
Civil status			
* Single, divorced or widowed*	13 (25%)	13 (25%)	26 (25%)
* Married-cohabiting*	38 (75%)	39 (75%)	77 (75%)
Educational status			
* Lower secondary or less*	16 (31%)	20 (38%)	36 (35%)
* Training system-vocational school (2-3 years)*	12 (24%)	5 (10%)	17 (17%)
* Upper secondary (4-5 years)*	18 (35%)	17 (33%)	35 (34%)
* University*	5 (10%)	10 (19%)	15 (15%)
Years from diabetes diagnosis	14 (9, 17)	13 (9, 17)	13 (9, 17)
BMI (Kg/cm2)	29.4 (25.6, 32.7)	26.7 (25.1, 30.7)	28.1 (25.3, 32.3)
sPA, mmHg	128 (119, 134)	127 (120, 136)	128 (120, 135)
dPA, mmHg	73 (70, 79)	72 (70, 80)	72 (70, 80)
HR, beats per minute	75 (66, 80)	76 (68, 83)	75 (66, 80)
Smoking habits			
* Former smoker*	20 (39%)	21 (40%)	41 (40%)
* No*	21 (41%)	23(44%)	44 (43%)
* Yes*	10 (20%)	8 (15%)	18 (17%)
Fasting blood glucose, mg/dl	141 (120, 167)	155 (130, 169)	147 (127, 168)
HbA1c, mmol/mol	60 (57, 68)	63 (59, 70)	62 (58, 70)
Cholesterol Total, mg/dl	147 (128, 187)	147 (127, 179)	147 (127, 185)
Cholesterol HDL, mg/dl	46 (43, 55)	43 (39, 58)	46 (39, 57)
Cholesterol LDL, mg/dl	71 (56, 104)	65 (53, 99)	67 (54, 102)
Cholesterol non-HDL, mg/dl	95 (76, 135)	99 (81, 133)	98 (77, 134)
Triglycerides, mg/dl	124 (88, 172)	125 (107, 198)	125 (97, 178)
Creatinine, mg/dl	0.91 (0.73, 1.00)	0.91 (0.80, 1.03)	0.91 (0.76, 1.01)
eGFR, ml/min	84 (72, 93)	85 (70, 94)	84 (70, 93)
ACR, mg/g	7 (5, 42)	20 (9, 49)	14 (6, 46)
Neuropathy^2^	12 (26%)	6 (13%)	18 (19%)
Missing data	5	5	10
Myocardial ischemia	9 (18%)	8 (15%)	17 (17%)
Heart failure	4 (7.8%)	2 (3.8%)	6 (5.8%)
Ischaemic cardiopathy	10 (20%)	10 (19%)	20 (19%)
Cerebrovascular disease	2 (3.9%)	2 (3.8%)	4 (3.9%)
Questionnaires			
* SF – PCS-12 *	50 (42, 54)	51 (42, 54)	50 (42, 54)
* SF – MCS-12 *	54 (46, 59)	51 (45, 56)	53 (35, 58)
* MMAS-8*	8.00 (6.00, 8.00)	7.38 ( 6.38, 8.00)	8.00 (6.75, 8.00)
* IPAQ (Mets)*	3810 (1050, 7080)	1548 (380, 3248)	2450 (653, 5145)
* DASI (score)*	50 (31, 58)	46 (35, 58)	47 (42, 58)
* DASI (Mets)*	8.91 (6.61, 9.89)	8.42 (7.01, 9.89)	8.51 (6.72, 9,89)

**^1^** n(%), median (interquartile range); ); **^2^** variable not imputed due to a high proportion of missing values. BMI, body mass index; IQR, interquartile range, sPA, systolic blood pressure; dPA, diastolic blood pressure; HbA1c, haemoglobin glycated; HDL, high-density lipoprotein; LDL, low-density lipoprotein; eGFR, Glomerular filtration rate (estimated by using the CKD-EPI formula); ACR, albumine/creatinine ratio in urines; SF – PCS-12, Short Form (Quality of life questionnaire) physical component score - 12; SF – MCS-12, Short Form (Quality of life questionnaire) mental component score – 12; MMAS-8, Morisky Medication Adherence Scale in the 8-item version; IPAQ, Physical Activity Questionnaire Daily; DASI, Duke Activity Status Index.

The median age at study entry was 67 years old (IQR: 59-72), with a median time from diabetes diagnoses to enrolment of 13 years (IQR: 9-17). Most participants were male (72%), married (75%), and held a secondary school degree (34%). Approximately 60% were current or former smokers. A significant proportion of participants reported comorbidities associated with diabetes, including ischemic heart disease, neuropathy, myocardial ischemia, and chronic kidney disease.

The median baseline fasting blood glucose level was 147 mg/dL (IQR: 127 to 168 mg/dl), with a glycated haemoglobin of 62 mmol/mol (IQR: 58 to 68 mmol/mol).

Regarding diabetes treatment, 96 participants (93%) were treated with metformin, 48 (47%) with sodium-glucose co-transporter type 2 (SGLT2) inhibitors, 34 (33%) with glucagon-like peptide-1 (GLP-1) receptor agonists, and 34 (33%) with basal insulin (**Supplementary Data Sheet S1**-**Supplementary Table S1**).

The baseline questionnaire scores (**Table 1**) suggest that, overall, participants reported moderate to good levels of physical activity, medication adherence, and quality of life, with some variability observed across individuals.

The results obtained, considering both PP and AT approaches, are entirely consistent with those obtained in the ITT analysis (**Supplementary Data Sheet S1—Supplementary Tables S2**-**S5**).

### 3.2 Number analysed

The number of participants included in the statistical analyses varied according to the population used: ITT, PP and AT:

- ITT population. 103 participants were included in this population: 51 in the intervention group and 52 in the control group
- PP population. 94 participants were included: 44 in the intervention group and 52 in the control group. Seven participants from the intervention group were excluded from the analysis due to violation of the study protocol. One participant failed to install the app on their smartphone, while six did not meet the minimum app usage threshold (72 entries per year, arbitrarily set). Additionally, two participants from the control group requested to discontinue participation.
- AT population. 103 participants were included: 44 in the intervention group and 59 in the control group. Seven participants not using the app as planned were reassigned to the control group.

### 3.3 Outcomes

#### 3.3.1 Primary outcome: change in HbA1c levels at 12 months

As reported in **Table 2**, both the intervention and control groups showed a significant decrease in HbA1c values 12 months after study enrolment (p < 0.001 for both groups). In the intervention group, HbA1c decreased from 60 mmol/mol (IQR: 57 to 68 mmol/mol) to 53 mmol/mol (IQR: 49 to 59 mmol/mol), showing a median change of -6 mmol/mol (IQR: -12 to -4 mmol/mol). In the control group, HbA1c decreased from 63 mmol/mol (IQR: 59 to 70 mmol/mol) to 55 mmol/mol (IQR: 50 to 64 mmol/mol), showing a median change of -7 mmol/mol (IQR: -14 to 0 mmol/mol). The median changes in HbA1c were similar between the two groups, with no statistically significant difference (p > 0.9).

**Table 2:** Baseline, 12-month, and changes (Δ) between the two time points in HbA1c levels, by intervention and control groups.

Group	Baseline^1^	T4^1^	p-value^2^	Δ (T4- Baseline)^1^	p-value^3^
HbA1c, mmol/mol					>0.9
Intervention	60 (57, 68)	53 (49, 59)*	<0.001	-6 (-12, -4)	
Control	63 (59,70)	55 (50, 64)*	<0.001	-7 (-14, 0)	

^1^Median (interquartile range); ^2^Wilcoxon signed-rank test comparing HbA1c at baseline and at T4; ^3^Wilcoxon rank sum test comparing haemoglobin glycated changes between the two groups; *Missing data—one for the intervention group two one for the control—were imputed using the multiple imputation by chained equations (MICE) algorithm. T4, 12 months; Δ, Difference between baseline and follow-up at 12; HbA1c, glycated haemoglobin.

To assess the impact of missing value imputation, a sensitivity analysis with different scenarios and different approaches (PP and AT) were performed (**Supplementary Data Sheet S2**, **Supplementary Tables SS1**, **SS4**, **SS5**, **SS8**, **SS9**, **SS12**, **SS13**, **SS16**, **SS19**, **SS22**, **SS25**, **SS26**, **SS29**, **SS30**, **SS33**, **SS34**, **SS37**, **SS38**, **SS41**). All analysis confirmed the previous findings.

#### 3.3.2 Secondary outcomes

##### 3.3.2.1 Clinical parameters and therapeutic target assessment

From the start to the completion of the study, no statistically significant between-group differences were observed in body weight variation (-2.6 kg; IQR: -5.0 to 0.2 vs. -3.0 kg; IQR: -5.1 to -0.6, p=0.8), LDL cholesterol variation (-23 mg/dl; IQR: -55 to -4 vs. -20 mg/dl; IQR: -55 to 3, p>0.9), non-HDL cholesterol variation (-8 mg/dl; IQR: -36 to 2 vs. -11 mg/dl; IQR: -30 to 8, p=0.7), sBP variation (1 mmHg; IQR: -10 to 8 vs. 5 mmHg; IQR: -16 to 8, p=0.3), and dBP variation (0 mmHg; IQR: -5 to 5 vs. 0 mmHg; IQR: -8 to 9, p>0.9) (**Table 3**). The percentage of participants whose HbA1c values fell below the threshold of 53 mmol/mol did not differ significantly between the groups (IG: 52% vs CG 42%, p = 0.4) (**Table 4**). The percentage of individuals attaining targets was comparable between the two groups for hypoglycaemic episodes (2.0% vs 7.7%, p=0.4), lipid target (71% vs 63%, p=0.4), BP target (75% vs 73%, p=0.9). Similarly, the percentage achieving the composite target was also comparable (25% vs 19%, p=0.4) (**Table 4**).

**Table 3:** Change in clinical feature values between the start and end of the study.

Difference between baseline and follow-up at 12 months	Intervention^1^ N = 51	Control^1^ N = 52	p-value^2^
Weight, Kg	-2.6 (-5.0, 0.2)*	-3.0 (-5.1, -0.6)*	0.8
Cholesterol LDL, mg/dl	-23 (-55, -4)*	-20 (-55, 3)*	>0.9
Cholesterol non-HDL, mg/dl	-8 (-36, 2)*	-11 (-30, 8)*	0.7
sBP, mmHg	-1 (-10, 8)*	-5 (-16, 8)*	0.3
dBP, mmHg	0 (-5, 5)*	0 (-8, 9)*	>0.9

^1^Median (interquartile range); ^2^Wilcoxon rank-sum test; *Missing data were imputed using the multiple imputation by chained equations (MICE) algorithm.

Δ, Difference between baseline and follow-up at 12 months; Kg, kilograms; LDL, low-density lipoprotein; HDL, high-density lipoprotein; sBP, systolic blood pressure; dBP, diastolic blood pressure.

**Table 4:** Percentage of patients who achieved the therapeutic target for the parameters of interest.

Variable	Baseline			T4		
	Intervention N = 51^1^	Control N = 52^1^	p-value^2^	Intervention N = 51^1^	Control N = 52^1^	p-value^2^
HbA1c target	0 (0%)	0 (0%)	–	26 (51%)	22 (42%)	0.4
Hypoglycaemic episodes	–	–	–	1 (2.0%)	4 (7.7%)	0.4
Lipid target	23 (45%)	25 (48%)	0.8	36 (71%)	33 (63%)	0.4
BP target	36 (71%)	37 (71%)	>0.9	38 (75%)	38 (73%)	0.9
Combined target: HbA1c + lipid + BP	–	–	–	13 (25%)	10 (19%)	0.4

^1^number (%); ^2^Pearson’s Chi-squared test; Fisher’s exact test;

HbA1c, haemoglobin glycated; BP, blood pressure; T4, 12 months.

hypoglycaemia is defined as a blood glucose level below 70 mg/dl or symptoms suggestive of it (e.g. shaking, sweating, dizziness); no baseline participants had a target HbA1c level (according to eligibility criteria).

##### 3.3.2.2 Assessment of questionnaire scores

**Table 5** presents scores from questionnaires administered at baseline and study completion.

**Table 5:** Baseline, 12-month, and changes (Δ) between the two time points in HbA1c levels.

	Baseline			T4			(T4-Baseline)		
Questionnaire	Intervention^1^ N = 51	Control^1^ N = 52	p-value^2^	Intervention^1^ N = 51	Control^1^ N = 52	p-value^2^	Intervention^1^ N = 51	Control^1^ N = 52	p-value^2^
SF – PCS-12	50 (42, 54)	51 (42, 54)	0.7	51 (44, 54)	49 (42, 54)	0.6	0 (-2, 5)	1 (-4, 4)	0.6
SF – MCS-12	54 (46, 59)	51 (45, 56)	0.042	54 (47, 58)	51 (46, 56)	0.20	-4 (-9, 2)	-4 (-6, 4)	0.6
MMAS-8	8.00 (6.00, 8.00)	7.38 (6.38, 8.00)	0.4	8.00 (6.63, 8.00)	8.00 (7.00, 8.00)	0.8	0.00 (0.00, 0.25)	0.00 (0.00, 1.00)	0.4
IPAQ (Mets)	3810 (1050, 7080)	1548 (380, 3248)	0.001	1890 (585-4545)	1740 (653, 4275)	0.8	-470 (-2980, 492)	283 (-571,1249)	0.022
DASI (score)	50 (31, 58)	46 (35, 58)	0.8	45 (30, 58)	46 (26, 58)	>0.9	0 (-5, 1)	0 (-10, 1)	0.7
DASI (Mets)	8.91 (6.61, 9.89)	8.42 (7.01, 9.89)	0.7	8,23 (6,44, 9.89)	8.42 (6.53, 9.89)	0.7	0.00 (-0.66, 0.14)	0.00 (-1.04, 0.16)	>0.9

^1^Median (interquartile range); ^2^Wilcoxon rank-sum test;

T4, 12 months; Δ, Difference between baseline and follow-up at 12; SF – PCS-12, Short Form (Quality of life questionnaire) physical component score SF-PCS- 12; SF – MCS-12, Short Form (Quality of life questionnaire) mental component score – 12; MMAS-8, Morisky Medication Adherence Scale in the 8-item version; IPAQ, Physical Activity Questionnaire Daily; DASI, Duke Activity Status Index.

The SF-12 physical domain (SF-PCS-12) showed similar results between groups at both time points. For the mental domain (SF-MCS-12), although the intervention group had a higher baseline score, this difference was no longer observed by month 12.

The MMAS-8 questionnaire^2^ indicated high medication adherence, with similar scores between the two groups at both time points.

The DASI questionnaire showed comparable potential physical activity between groups at both baseline and study completion.

Although the IPAQ questionnaire showed a significant difference between groups in baseline scores, this was substantially reduced by study end with a greater decrease in the intervention group.

At 12 months, a questionnaire assessing app usability was administered exclusively to the intervention group. Of the 51 participants, 42 responded to the SUS questionnaire, achieving a median score of 95 (IQR: 78 to 99).

##### 3.3.2.3 Clinical and biohumoral data across Time points 1 through 4

Clinical and biohumoral data measured across the 4 follow-up points were reported in **Supplementary Table S6** (**Supplementary Data Sheet S1**). Due to extensive missing data, imputation was not possible. No significant differences were observed between groups in the incidence of hypoglycaemic episodes, urgent metabolic, cardiovascular, cerebrovascular, nephrological consultations, hospitalisations (0 events across T1-T4), or therapeutic changes (median < 2), which were comparable between groups.

##### 3.3.2.4 Application data usage

**Figure 3:**
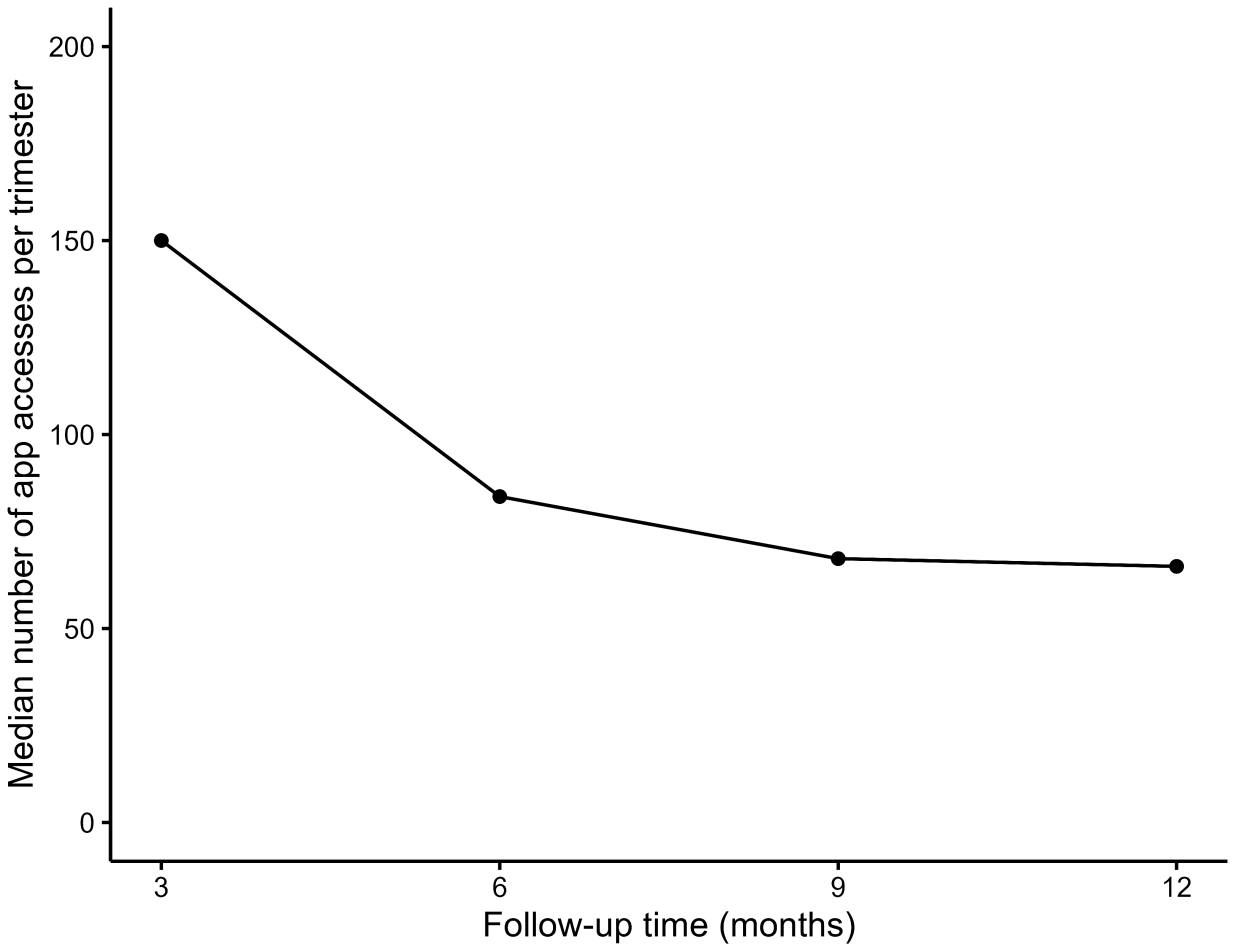
Median frequency of data entry every 4 months over12 months by the intervention group.

The graph (**Figure 3**) below shows that the median frequency of data entry in the app significantly decreased from the first to the second quarter, after which it stabilised (median frequency at T1: 150; IQR: 52 to 444, T2: 84; IQR: 37 to 423, T3: 68; IQR: 19 to 449, T4: 66; IQR: 9 to 413, p=0.001).

The results of the exploratory *post-hoc* analysis, examining the three domains A, B, and C as described in the methods paragraph, were provided in the **Supplementary Materials** (**Supplementary Data Sheet S2**, **Supplementary Table S7**).

Sensitivity analysis with three different scenarios, as well as the PP and AT analyses, corroborates previous findings (**Supplementary Data Sheet S2**, **SS2**, **SS3**, **SS6**, **SS7**, **SS10**, **SS11**, **SS14**, **SS15**, **SS17**, **SS18**, **SS20**, **SS21**, **SS23**, **SS24**, **SS27**, **SS28**, **SS31**, **SS32**, **SS35**, **SS36**, **SS39**, **SS40**).

### 3.4 Harms

During the study, there were two significant interruptions to the “TreC Diabete” platform. No issues related to the handling of personal data occurred.

## 4 Discussion

### 4.1 Limitations

Several technical and operational issues related to the use of the platform emerged:

1. Outdated WebView and app startup issues: some participants couldn’t use the app due to an outdated WebView, affecting the user experience and causing a participant to be assigned to the control group.
2. Complex profile creation: the process involved multiple steps (e.g. entering social security number, time-sensitive passwords), which cause difficulties, especially when switching devices.
3. Service interruptions: two network outages (2 and 12 days) disrupted communication and data access, requiring alternative communication methods like email and phone.
4. Data entries anomalies: incorrect data entries were only fixable when reinstalling the app, leading to lost time and resources.

Other limitations not directly related to the platform were:

1. Telemedicine visits time assessment: data on time spent on telemedicine visits at 6 months was not collected, as visits followed standardised timelines not aligned with study requirements.
2. Limited staff availability: involving medical and nursing staff was challenging and therefore, the PI conducted some follow-up visits, deviating from the protocol.
3. Routine activity versus study protocol: misalignment between routine clinic activities and study timelines led to some visits being scheduled closer together than originally intended, potentially altering the flow of visits.
4. Data collection via phone: telephone contact was used on a quarterly basis due to limited data retrieval methods, resulting in more interactions than planned, which may have influenced results.
5. Smaller sample size than anticipated: slow enrolment resulted in an initial smaller sample size than planned (103 out of 120 participants). However, the under-recruitment did not impact the statistical power, which was set at 80%, and the significant level at %5 for the primary outcome
6. Possible dilution of intervention effect: despite the researchers’ effort to minimise and standardised interactions with participants in the control group, this is a possibility that these interactions could have influenced the outcomes, reducing the likelihood of detecting a significant difference between the control group and the intervention group.
7. Data issues:
  - Missing data: three missing data for the primary endpoint and a higher number of missing data for the secondary endpoint weakened the ability to assess intervention efficacy. To mitigate these issues, several statistical analyses were conducted.
  - Low number of events for secondary outcomes: events, such as specialist visits or hospitalisations, were insufficient to evaluate the intervention’s impact. A larger number of events and or number of subjects or longer follow-up period are necessary.

### 4.2 Generalisability

An internal analysis using secondary data from the PAT population and The Johns Hopkins Adjusted Clinical Group System (The Johns Hopkins ACG^®^ System) version 11.2 (29) revealed that the Telemechron study sample was slightly younger (median age 67) than the T2DM PAT population (median age 70). The trial population had a higher percentage of males (71.8%) compared to the PAT population (56.4%), with a more pronounced sex gap in the 50–69 age range, while the 70–85 age group showed a more balanced distribution. The observed imbalance in the study population concerning sex and age distribution is supported by both epidemiological data and the specific eligibility criteria employed. Epidemiologically, in Italy, type 2 diabetes prevalence exhibits a gender shift across age groups (30). Younger individuals with type 2 diabetes are predominantly male, while women are more represented in the population over seventy-five years old. Consequently, the relatively younger average age of the study participants likely contributed to the higher enrolment of male subjects. Furthermore, the study’s eligibility criteria played a significant role in shaping the demographic profile of the recruited patients. The exclusion of individuals who required walking aids, lacked smartphone access, presented with advanced medical complications or reduced life expectancy, or were unwilling or unable to utilize mobile technology inherently favoured the inclusion of individuals who were generally younger. These criteria inadvertently created a selection bias, and the study results may be more applicable to younger male individuals in the province’s diabetic population.

Another limitation is that participants were required to have HbA1c levels between 53 mmol/mol (7%) and 108 mmol/mol (12%), while over 50% of the diabetic population in PAT had HbA1c levels below 53 mmol/mol. Therefore, the findings mainly apply to individuals with non-target HbA1c levels, which were the subject of the study.

Additionally, the study’s adherence and technology use may not reflect real-world practices, as participants were monitored more frequently than in standard outpatient care. Despite positive feedback on the system’s usability, the significant decrease in app usage over time suggests that long-term engagement with the “TreC Diabete” platform may be challenging.

### 4.3 Main results

Our trial primarily aimed to evaluate the “TreC Diabete” platform impact by comparing HbA1c levels between two groups of patients with T2DM referred to provincial diabetes centres. Additionally, secondary objectives included the evaluation of other health outcomes and several process indicators.

At 12 months post-randomization, the change in median HbA1c levels did not differ significantly between the two groups. Therefore, there is no evidence to suggest that the platform influenced the reduction in HbA1c levels in the intervention group compared to the control group. Similarly, secondary outcomes also showed no statistically or clinically significant differences between the two groups. However, the overall reduction in HbA1c levels from baseline to the end of the study was substantial, with 51% of the intervention group and 42% of the control group achieving the glycaemic target (HbA1c < 53 mmol/mol). These findings were consistent across various analyses, including sensitivity models.

Regarding app uses, data entries decreased significantly from the first quarter to the second quarter but subsequently stabilised (p = 0.001). Most participants in the intervention group adhered well to their clinician’s prescriptions, except for the daily treatment records, which consistently showed low completion rates. Only 2% of users confirmed their medication was taken, although this information does not align with the medication adherence reported in the MMAS-8 questionnaires^3^. System usability, as assessed by 42 participants in the intervention group out of 52, using the SUS questionnaire, had a median score of 95 (range: 0–100), indicating a high level of satisfaction with the platform. Patient self-reporting of therapy intake is likely inaccurate due to the effort required. In fact, both the satisfaction questionnaire and the improvement in clinical and biochemical parameters dependent on consistent therapy intake support adequate treatment adherence. It is not possible to assess whether the reminder to take the therapy may have positively influenced treatment adherence. Future research should explore more reliable methods for confirming therapy intake.

### 4.4 Comparison with other studies

There has been a notable surge in studies assessing the clinical efficacy of mobile applications, especially concerning the management of chronic diseases. These reviews delve into various aspects, including user perspectives and healthcare provider engagement.

Studies such as the 2023 meta-synthesis of 9 qualitative studies by Creber and colleagues, aiming to explore barriers and facilitators of telemonitoring devices for adults with chronic diseases, highlighted 4 key themes: Improved Care, Communication, Technology Acceptability and Feasibility and Intervention Concerns (31). Authors suggested that users generally perceive these devices as acceptable and feasible within a short-medium timeframe, although difficulties in using technologies due to a generational gap exist. These challenges may be overcome with targeted education and support. The acceptability and effectiveness of these devices in the short-medium term are also supported by other reviews focusing on various chronic conditions.

Among reviews concerned with diabetes and mHealth system, a meta-analysis study by de Souza Ferreira and colleagues, including 17 trials on diabetes highlighted the effectiveness of mobile apps for monitoring diabetes and hypertension, showing a 0.39% (95% CI 0.24 to 0.54) reduction in HbA1c compared to traditional care (32). Similarly, the meta-analysis conducted by Lee and colleagues including 16 RCTs (participants = 6,204) and focusing on older adults with diabetes, showed several improvements in glycaemic outcomes with a pooled mean HbA1c decreased of -0.24% (95% CI -0.44 to -0.05) (33). Furthermore, a 2024 meta-analysis from Liu and colleagues, incorporating data from 1,456 patients and different telemedicine solutions, confirmed that telemedicine significantly enhanced self-management and lowered blood glucose levels in T2DM patients (7). Key improvements were also seen in HbA1c, two-hours post-prandial glucose, weight, SBP, and DBP, but not on BMI and Fasting Plasma Glucose. Subgroup analyses revealed the greatest efficacy in younger patients, interventions under six months, and the use of apps and telephones. Further large-scale studies are needed to address existing limitations.

The literature presents conflicting findings on the impact of apps in improving therapy adherence (34) (35). Our study demonstrated excellent adherence, as measured by the MMAS-8 questionnaire^4^ and improvements in biohumoral parameters, despite a minimal percentage of therapy intake confirmations recorded in the app. While it is unclear whether the reminder function enhanced adherence, it is evident that the request to log therapy intake was largely disregarded. This issue warrants reconsideration, with alternative approaches to be explored in future studies.

In diabetes, the use of telemedicine must be harmonised with the in-person visit, to collect the clinical elements necessary for the correct disease management and resource optimisation. Telemedicine, used as the sole approach to T2DM treatment, is less effective than combined use with an in-person visit or in-person visit alone for glycaemic control, especially in those patients with a worse metabolic control and a complex treatment (36). Telemedicine may be able to achieve similar glycaemic control while potentially saving resources, making it a positive outcome.

### 4.5 Psychosocial aspects

The integration of digital health solutions like the “TreC Diabete” platform into chronic disease management extends beyond clinical outcomes, addressing psychological and social dimensions. While the trial showed limited improvements in HbA1c levels, it highlighted key psychosocial aspects influencing patient engagement and well-being.

Recruiting older participants was challenging, primarily due to smartphone ownership requirements, digital literacy and participation commitments, underscoring the digital divide effect (37). Despite initial registration and technical issues, most participants actively used the app, reflected in a high SUS score (median 95/100), indicating positive user satisfaction and motivation. Patients engaged by entering clinical data and using the chat feature to communicate with healthcare providers, demonstrating the platform’s potential for self-management support. However, data entry rates declined after the first quarter, and adherence to medication intake confirmation remained low (2.0%), suggesting that mHealth tools enhance only certain aspects of self-management. This “engagement fatigue” highlights the need for sustained motivation strategies, such as personalized feedback, gamification, social support features, or structured telemonitoring sessions (38). The burden of daily manual data entry may discourage users, while frequent reminders can become overwhelming, leading to notification dismissal. Alternative medication reporting methods, such as weekly summaries or input only when a dose is missed, could be explored (38).

Despite the high SUS score, the low adherence to therapy intake confirmation may reflect a discrepancy between perceived usability and the actual burden of continuous manual input. Participants might have viewed the platform primarily as a communication and information tool rather than a strict tracking system. This distinction suggests that usability alone does not guarantee sustained engagement with all app functionalities. Further investigation is needed to identify barriers to reporting behaviours and to differentiate between satisfaction with interface design and long-term behavioural compliance.

The chat feature’s significant use (78.4% of participants) underscores the role of mHealth in fostering connectivity and reducing isolation. This aligns with Self-Determination Theory, which emphasizes competence, autonomy, and relatedness in chronic disease management (39) (40) (41).

Unlike other studies that have examined mHealth tools in a fragmented manner—focusing separately on televisits, educational interventions, or patient self-managed clinical diaries (8)—”TreC Diabete” stands out as a unique case. It integrates these key features into a single, unified platform, offering a comprehensive digital health solution. Grounded in theoretical frameworks like Self-Determination Theory and the Technology-Enabled Self-Management feedback loop (42), this holistic approach enhances mHealth interventions by addressing multiple aspects of chronic disease management simultaneously and providing a mix between face-to-face visits and telemedicine. This convergence of functionalities underscores the importance of considering both technical and psychosocial factors to foster an advanced patient-centred digital health strategy.

### 4.6 Recommendation

Several recommendations can be made for future research and clinical practice:

- Ensuring automatic data transfer between the instruments used to capture clinical parameters and the technology platform could significantly improve the accuracy and completeness of recorded data. This approach would reduce the burden of manual data entry on patients and minimise the risk of transcription errors.
- Forthcoming studies focusing on telemedicine systems should actively involve healthcare professionals and patients from the earliest stages of development; such collaboration could enhance system usability, ensure that the technology aligns with clinical workflows, and address patient needs more effectively.
- Future studies should consider a longer follow-up period or a larger patient cohort to be able to assess not only long-term clinical outcomes (such as hospitalisation) but also the adherence to telemedicine-related intervention.
- Randomised controlled trials with a specific focus on telemedicine systems should place greater emphasis on assessing resource utilization by both health systems and patients. This would provide insights into cost-effectiveness, time savings, and the overall efficiency of telemedicine interventions.

## 5 Conclusion

In conclusion, the “TreC Diabete” platform did not improve glycaemic control and other clinical and biohumoral parameters compared to the control group in a frame time of one year. However, the study highlighted key factors for future implementations, such as identifying patients who will benefit most from telemedicine and optimising platform use, thereby enhancing adherence between patients and healthcare staff. Successful integration into clinical practice requires understanding the benefits, addressing adoption barriers, and providing education and support to both patients and healthcare providers, ultimately ensuring equitable healthcare delivery. Finally, our study results concur along with existing evidence, contributing to the understanding of how mHealth can be integrated into healthcare organisational models.

## 6 Other information

### 6.1 Study registration

The study was registered on ClinicalTrials.gov on 11/29/2022 (NCT05629221).

### 6.2 Protocol

The study methods are detailed in protocol version n.02 dated 09 August 2022, titled “Organizational models supported by technology for the management of diabetic disease and its complications in the diabetes clinic setting. A randomized controlled trial in patients with type 2 diabetes mellitus in less-than-ideal glycaemic control.”. The protocol was also published in *Trials BMC* under the title “Organisational models supported by technology for the management of diabetic disease and its complications in a diabetic clinical setting: study protocol for a randomized controlled trial targeting type 2 diabetes individuals with non -ideal glycaemic values (Telemechron study) and is accessible at this link: https://trialsjournal.biomedcentral.com/articles/10.1186/s13063-023-07515-6 (11).

## Data Availability

The raw data supporting the conclusions of this article cannot be shared publicly due to the presence of personal health information and the restrictions outlined in the informed consent form obtained from participants. However, data may be made available from the authors upon reasonable request, subject to compliance with applicable data protection regulations (including the GDPR) and the relevant ethical approvals.

## Footnotes

1 The MMAS-8 Scale (U.S. Copyright Registration No. TX0008632533), content, name, and trademarks are protected by US copyright and trademark laws. Permission for use of the scale and its coding is required. A license agreement is available from MMAR, LLC., www.moriskyscale.com.

2 The MMAS-8 Scale (U.S. Copyright Registration No. TX0008632533), content, name, and trademarks are protected by US copyright and trademark laws. Permission for use of the scale and its coding is required. A license agreement is available from MMAR, LLC., www.moriskyscale.com.

3 The MMAS-8 Scale (U.S. Copyright Registration No. TX0008632533), content, name, and trademarks are protected by US copyright and trademark laws. Permission for use of the scale and its coding is required. A license agreement is available from MMAR, LLC., www.moriskyscale.com.

4 The MMAS-8 Scale (U.S. Copyright Registration No. TX0008632533), content, name, and trademarks are protected by US copyright and trademark laws. Permission for use of the scale and its coding is required. A license agreement is available from MMAR, LLC., www.moriskyscale.com.
