# Supplementary material for: Impact of digital health on Type 2 diabetes management: a randomised controlled trial of the ‘TreC Diabete’ platform (TELEMECHRON Study)
Source: Front Clin Diabetes Healthc. 2025 Jun 10;6:1589548. doi: 10.3389/fcdhc.2025.1589548 (PMC12186056; doi:10.3389/fcdhc.2025.1589548)
Supplement: Supplementary file 1 [file DataSheet1.docx]

**Table S1.** Current medications at baseline by allocation group and total group, using the Intention-To-Treat (ITT) approach.

| **Variable** | **Total**  **N = 103^1^** | **Intervention**  **N = 51^1^** | **Control**  **N = 52^1^** |  |
| --- | --- | --- | --- | --- |
| Diet only | 4 (3.9%) | 2 (3.9%) | 2 (3.8%) |  |
| Biguanides (Metformin) | 96 (93%) | 47 (92%) | 49 (94%) |  |
| Sulphonylureas and glinides | 6 (5.8%) | 1 (2.0%) | 5 (9.6%) |  |
| Dipeptidyl peptidase-4 (DPP-4) inhibitors | 15 (15%) | 5 (9.8%) | 10 (19%) |  |
| Glucagon-like peptide-1 (GLP-1) analogues | 34 (33%) | 14 (27%) | 20 (38%) |  |
| Glyflozines - Inhibitors of sodium-glucose co-transporter type 2 (SGLT2) | 48 (47%) | 25 (49%) | 23 (44%) |  |
| Thiazolidinediones (Pioglitazone) | 3 (2.9%) | 1 (2.0%) | 2 (3.8%) |  |
| Alpha-glucosidase inhibitors (Acarbose) | 1 (1.0%) | 0 (0%) | 1 (1.9%) |  |
| Intermediate or long acting insulin | 34 (33%) | 18 (35%) | 16 (31%) |  |
| Fast-acting insulin | 5 (4.9%) | 4 (7.8%) | 1 (1.9%) |  |
| ACE-inhibitors or sartans | 66 (64%) | 30 (59%) | 36 (69%) |  |
| Angiotensin/neprilysin receptor inhibitors (ARNI) | 1 (1.0%) | 0 (0%) | 1 (1.9%) |  |
| Beta-blockers | 37 (36%) | 20 (39%) | 17 (33%) |  |
| Calcium channel blockers | 32 (31%) | 16 (31%) | 16 (31%) |  |
| Loop diuretics | 8 (7.8%) | 5 (9.8%) | 3 (5.8%) |  |
| Thiazide diuretics | 23 (22%) | 10 (20%) | 13 (25%) |  |
| Mineralocorticoid receptor antagonists (MRA) | 3 (2.9%) | 1 (2.0%) | 2 (3.8%) |  |
| Other anti-hypertensive drugs | 0 (0%) | 0 (0%) | 0 (0%) |  |
| Antiplatelet agents | 43 (42%) | 18 (35%) | 25 (48%) |  |
| Oral anticoagulants | 7 (6.8%) | 4 (7.8%) | 3 (5.8%) |  |
| Statins | 77 (75%) | 36 (71%) | 41 (79%) |  |
| Ezetimibe | 35 (34%) | 14 (27%) | 21 (40%) |  |
| Fibrates | 4 (3.9%) | 1 (2.0%) | 3 (5.8%) |  |
| Other anti-lipid medications | 12 (12%) | 7 (14%) | 5 (9.6%) |  |
| Other cardiovascular medications | 7 (6.8%) | 5 (9.8%) | 2 (3.8%) |  |
| **^1^**n(%). | | | | |

**Table S2** Baseline demographics, clinical, anamnestic, biohumoral characteristics, and questionnaire scores, presented by allocation group and the total group, using the Per-Protocol (PP) approach.

| **Variable** | **Total**  **N = 94^1^** | **Intervention**  **N = 44^1^** | **Control**  **N = 50^1^** |
| --- | --- | --- | --- |
| Sex |  |  |  |
| *Female* | 24 (26%) | 14 (32%) | 10 (20%) |
| *Male* | 70 (74%) | 30 (68%) | 40 (80%) |
| Age (years) | 67 (59, 71) | 66 (61, 70) | 68 (59, 72) |
| Civil status |  |  |  |
| *Single, divorced or widowed* | 24 (26%) | 11 (25%) | 13 (26%) |
| *Married-cohabiting* | 70 (74%) | 33 (75%) | 37 (74%) |
| Educational status |  |  |  |
| *Lower secondary or less* | 34 (36%) | 15 (34%) | 19 (38%) |
| *Training system-vocational school (2-3 years)* | 15 (16%) | 10 (23%) | 5 (10%) |
| *Upper secondary (4-5 years)* | 30 (32%) | 14 (32%) | 16 (32%) |
| *University* | 15 (16%) | 5 (11%) | 10 (20%) |
| Years from diabetes diagnosis | 13 (9, 17) | 14 (10, 17) | 13 (9, 17) |
| BMI (Kg/cm2) | 27.9 (25.3, 32.1) | 29.1 (25.5, 32.2) | 27.1 (25.3, 31.3) |
| sPA, mmHg | 128 (119, 134) | 128 (118, 132) | 125 (120, 135) |
| dPA, mmHg | 72 (70, 80) | 72 (70, 78) | 71 (69, 80) |
| HR, beats per minute | 75 (66, 80) | 75 (66, 80) | 75 (67, 82) |
| Smoking habits |  |  |  |
| *Former smoker* | 38 (40%) | 18 (41%) | 20 (40%) |
| *No* | 40 (43%) | 17 (39%) | 23 (46%) |
| *Yes* | 16 (17%) | 9 (20%) | 7 (14%) |
| Neuropathy^2^  Missing data | 16 (19%)  10 | 10 (26%)  5 | 6 (13%)  5 |
| Myocardial ischemia | 15 (16%) | 8 (18%) | 7 (14%) |
| Heart failure | 5 (5.3%) | 3 (6.8%) | 2 (4.0%) |
| Ischaemic cardiopathy | 18 (19%) | 9 (20%) | 9 (18%) |
| Cerebrovascular disease | 4 (4.3%) | 2 (4.5%) | 2 (4.0%) |
| Fasting blood glucose, mg/dl | 150 (127, 168) | 142 (120, 167) | 155 (131, 174) |
| HbA1c, mmol/mol | 62 (57, 69) | 59 (56, 66) | 63 (59, 71) |
| Cholesterol Total, mg/dl | 146 (127, 177) | 145 (127, 169) | 150 (128, 182) |
| Cholesterol HDL, mg/dl | 46 (39, 57) | 48 (44, 56) | 43 (38, 57) |
| Cholesterol LDL, mg/dl | 66 (55, 99) | 64 (55, 97) | 67 (55, 99) |
| Cholesterol non-HDL, mg/dl | 95 (76, 128) | 91 (74, 119) | 99 (82, 134) |
| Triglycerides, mg/dl | 123 (96, 173) | 107 (87, 161) | 127 (110, 200) |
| Creatinine, mg/dl | 0.91 (0.77, 1.01) | 0.91 (0.73, 0.98) | 0.91 (0.80, 1.04) |
| eGFR, ml/min | 84 (71, 93) | 84 (75, 93) | 85 (69, 94) |
| ACR, mg/g | 14 (6, 46) | 7 (5, 41) | 20 (9, 50) |
| Questionnaires |  |  |  |
| *SF – PCS-12* | 50 (43, 54) | 50 (44, 54) | 51 (42, 55) |
| *SF – MCS-12* | 53 (44, 58) | 54 (45, 58) | 51 (44, 56) |
| *MMAS-8* | 8.00 (6.75, 8.00) | 8.00 (6.56, 8.00) | 7.75 (6.75, 8.00) |
| *IPAQ (Mets)* | 2243 (603, 4735) | 3,655 (724, 6578) | 1,548 (310, 3113) |
| *DASI (score)* | 50 (33, 58) | 51 (31, 58) | 46 (35, 58) |
| *DASI (Mets)* | 8.91 (6.77, 9.89) | 8.97 (6.61, 9.89) | 8.42 (7.01, 9.89) |
| **^1^**n(%), median (interquartile range); **^2^**variable not imputed due to a high proportion of missing values. **Abbreviations:** BMI = body mass index; IQR = interquartile range, sPA = systolic blood pressure; dPA = diastolic blood pressure; HbA1c = haemoglobin glycated; HDL = high-density lipoprotein; LDL = low-density lipoprotein; eGFR = Glomerular filtration rate (estimated by using the CKD-EPI formula [17]; ACR = albumine/creatinine ratio in urines; SF – PCS-12 = Short Form (Quality of life questionnaire) physical component score - 12; SF – MCS-12 = Short Form (Quality of life questionnaire) mental component score – 12; MMAS-8 = Morisky Medication Adherence Scale in the 8-item version; IPAQ = Physical Activity Questionnaire Daily; DASI = Duke Activity Status Index. | | | |

**Table S3.** Current medications at baseline by allocation group and total group, using the Per-Protocol (PP) approach.

| **Variable** | **Total**  **N = 94^1^** | **Intervention**  **N = 44^1^** | **Control**  **N = 50^1^** |  |
| --- | --- | --- | --- | --- |
| Diet only | 4 (4.3%) | 2 (4.5%) | 2 (4.0%) |  |
| Biguanides (Metformin) | 88 (94%) | 41 (93%) | 47 (94%) |  |
| Sulphonylureas and glinides | 6 (6.4%) | 1 (2.3%) | 5 (10%) |  |
| Dipeptidyl peptidase-4 (DPP-4) inhibitors | 14 (15%) | 4 (9.1%) | 10 (20%) |  |
| Glucagon-like peptide-1 (GLP-1) analogues | 33 (35%) | 14 (32%) | 19 (38%) |  |
| Glyflozines - Inhibitors of sodium-glucose co-transporter type 2 (SGLT2) | 41 (44%) | 20 (45%) | 21 (42%) |  |
| Thiazolidinediones (Pioglitazone) | 3 (3.2%) | 1 (2.3%) | 2 (4.0%) |  |
| Alpha-glucosidase inhibitors (Acarbose) | 1 (1.1%) | 0 (0%) | 1 (2.0%) |  |
| Intermediate or long acting insulin | 32 (34%) | 16 (36%) | 16 (32%) |  |
| Fast-acting insulin | 3 (3.2%) | 2 (4.5%) | 1 (2.0%) |  |
| ACE-inhibitors or sartans | 59 (63%) | 25 (57%) | 34 (68%) |  |
| Angiotensin/neprilysin receptor inhibitors (ARNI) | 1 (1.1%) | 0 (0%) | 1 (2.0%) |  |
| Beta-blockers | 34 (36%) | 18 (41%) | 16 (32%) |  |
| Calcium channel blockers | 28 (30%) | 13 (30%) | 15 (30%) |  |
| Loop diuretics | 7 (7.4%) | 4 (9.1%) | 3 (6.0%) |  |
| Thiazide diuretics | 23 (24%) | 10 (23%) | 13 (26%) |  |
| Mineralocorticoid receptor antagonists (MRA) | 3 (3.2%) | 1 (2.3%) | 2 (4.0%) |  |
| Other anti-hypertensive drugs | 94 (100%) | 44 (100%) | 50 (100%) |  |
| Antiplatelet agents | 41 (44%) | 18 (41%) | 23 (46%) |  |
| Oral anticoagulants | 6 (6.4%) | 3 (6.8%) | 3 (6.0%) |  |
| Statins | 71 (76%) | 32 (73%) | 39 (78%) |  |
| Ezetimibe | 32 (34%) | 13 (30%) | 19 (38%) |  |
| Fibrates | 4 (4.3%) | 1 (2.3%) | 3 (6.0%) |  |
| Other anti-lipid medications | 11 (12%) | 6 (14%) | 5 (10%) |  |
| Other cardiovascular medications | 7 (7.4%) | 5 (11%) | 2 (4.0%) |  |
| **^1^**n(%). | | | | |

**Table S4** Baseline demographics, clinical, anamnestic, biohumoral characteristics, and questionnaire scores, presented by allocation group and the total group, using the As-Treated (AT) approach.

| **Variable** | **Total**  **N = 103^1^** | **Intervention**  **N = 44^1^** | **Control**  **N = 59^1^** |
| --- | --- | --- | --- |
| Sex |  |  |  |
| *Female* | 29 (28%) | 14 (32%) | 15 (25%) |
| *Male* | 74 (72%) | 30 (68%) | 44 (75%) |
| Age (years) | 67 (59, 72) | 66 (61, 70) | 68 (59, 72) |
| Civil status |  |  |  |
| *Single, divorced or widowed* | 26 (25%) | 11 (25%) | 15 (25%) |
| *Married-cohabiting* | 77 (75%) | 33 (75%) | 44 (75%) |
| Educational status |  |  |  |
| *Lower secondary or less* | 35 (35%) | 15 (34%) | 21 (35%) |
| *Training system-vocational school (2-3 years)* | 17 (17%) | 10 (23%) | 7 (12%) |
| *Upper secondary (4-5 years)* | 35 (34%) | 14 (32%) | 21 (36%) |
| *University* | 15 (15%) | 5 (11%) | 10 (17%) |
| Years from diabetes diagnosis | 13 (9, 17) | 14 (10, 17) | 13 (9, 17) |
| BMI (Kg/cm2) | 28.1 (25.3, 32.3) | 29.1 (25.5, 32.2) | 27.2 (25.3, 32.2) |
| sPA, mmHg | 128 (120, 135) | 128 (118, 132) | 128 (120, 138) |
| dPA, mmHg | 72 (70, 80) | 72 (70, 78) | 74 (70, 80) |
| HR, beats per minute | 75 (66, 80) | 75 (66, 80) | 76 (68, 83) |
| Smoking habits |  |  |  |
| *Former smoker* | 41 (40%) | 18 (41%) | 23 (39%) |
| *No* | 44 (43%) | 17 (39%) | 27 (46%) |
| *Yes* | 18 (17%) | 9 (20%) | 9 (15%) |
| Neuropathy^2^  Missing data | 18 (19%)  10 | 10 (26%)  5 | 8 (15%)  5 |
| Myocardial ischemia | 17 (17%) | 8 (18%) | 9 (15%) |
| Heart failure | 6 (5.8%) | 3 (6.8%) | 3 (5.1%) |
| Ischaemic cardiopathy | 20 (19%) | 9 (20%) | 11 (19%) |
| Cerebrovascular disease | 4 (3.9%) | 2 (4.5%) | 2 (3.4%) |
| Fasting blood glucose, mg/dl | 147 (127, 168) | 142 (120, 167) | 154 (130, 168) |
| HbA1c, mmol/mol | 62 (58, 70) | 59 (56, 66) | 63 (59, 71) |
| Cholesterol Total, mg/dl | 147 (127, 185) | 145 (127, 169) | 151 (129, 188) |
| Cholesterol HDL, mg/dl | 46 (39, 57) | 48 (44, 56) | 43 (39, 57) |
| Cholesterol LDL, mg/dl | 67 (54, 102) | 64 (55, 97) | 67 (54, 106) |
| Cholesterol non-HDL, mg/dl | 98 (77, 134) | 91 (74, 119) | 105 (82, 142) |
| Triglycerides, mg/dl | 125 (97, 178) | 107 (87, 161) | 133 (109, 200) |
| Creatinine, mg/dl | 0.91 (0.76, 1.01) | 0.91 (0.73, 0.98) | 0.90 (0.79, 1.05) |
| eGFR, ml/min | 84 (70, 93) | 84 (75, 93) | 85 (69, 94) |
| ACR, mg/g | 14 (6, 46) | 7 (5, 41) | 19 (8, 49) |
| Questionnaires |  |  |  |
| *SF – PCS-12* | 50 (42, 54) | 50 (44, 54) | 51 (42, 54) |
| *SF – MCS-12* | 53 (45, 58) | 54 (45, 58) | 52 (45, 57) |
| *MMAS-8* | 8.00 (6.75, 8.00) | 8.00 (6.56, 8.00) | 7.75 (6.63, 8.00) |
| *IPAQ (Mets)* | 2,450 (653, 4,838) | 3945 (973, 6578) | 1980 (455, 3615) |
| *DASI (score)* | 47 (32, 58) | 51 (31, 58) | 45 (34, 58) |
| *DASI (Mets)* | 8.51 (6.72, 9.89) | 8.97 (6.61, 9.89) | 8.33 (6.96, 9.89) |
| **^1^**n(%), median (interquartile range); **^2^**variable not imputed due to a high proportion of missing values. **Abbreviations:** BMI = body mass index; IQR = interquartile range, sPA = systolic blood pressure; dPA = diastolic blood pressure; HbA1c = haemoglobin glycated; HDL = high-density lipoprotein; LDL = low-density lipoprotein; eGFR = Glomerular filtration rate (estimated by using the CKD-EPI formula [17]; ACR = albumine/creatinine ratio in urines; SF – PCS-12 = Short Form (Quality of life questionnaire) physical component score - 12; SF – MCS-12 = Short Form (Quality of life questionnaire) mental component score – 12; MMAS-8 = Morisky Medication Adherence Scale in the 8-item version; IPAQ = Physical Activity Questionnaire Daily; DASI = Duke Activity Status Index. | | | |

**Table S5.** Current medications at baseline by allocation group and total group, considering as-treated (AT) approach.

| **Variable** | **Total**  **N = 94^1^** | **Intervention**  **N = 44^1^** | **Control**  **N = 50^1^** |  |
| --- | --- | --- | --- | --- |
| Diet only | 4 (3.9%) | 2 (4.5%) | 2 (3.4%) |  |
| Biguanides (Metformin) | 96 (93%) | 41 (93%) | 55 (93%) |  |
| Sulphonylureas and glinides | 6 (5.8%) | 1 (2.3%) | 5 (8.5%) |  |
| Dipeptidyl peptidase-4 (DPP-4) inhibitors | 15 (15%) | 4 (9.1%) | 11 (19%) |  |
| Glucagon-like peptide-1 (GLP-1) analogues | 34 (33%) | 14 (32%) | 20 (34%) |  |
| Glyflozines - Inhibitors of sodium-glucose co-transporter type 2 (SGLT2) | 48 (47%) | 20 (45%) | 28 (47%) |  |
| Thiazolidinediones (Pioglitazone) | 3 (2.9%) | 1 (2.3%) | 2 (3.4%) |  |
| Alpha-glucosidase inhibitors (Acarbose) | 1 (1.0%) | 0 (0%) | 1 (1.7%) |  |
| Intermediate or long acting insulin | 34 (33%) | 16 (36%) | 18 (31%) |  |
| Fast-acting insulin | 5 (4.9%) | 2 (4.5%) | 3 (5.1%) |  |
| ACE-inhibitors or sartans | 66 (64%) | 25 (57%) | 41 (69%) |  |
| Angiotensin/neprilysin receptor inhibitors (ARNI) | 1 (1.0%) | 0 (0%) | 1 (1.7%) |  |
| Beta-blockers | 37 (36%) | 18 (41%) | 19 (32%) |  |
| Calcium channel blockers | 32 (31%) | 13 (30%) | 19 (32%) |  |
| Loop diuretics | 8 (7.8%) | 4 (9.1%) | 4 (6.8%) |  |
| Thiazide diuretics | 23 (22%) | 10 (23%) | 13 (22%) |  |
| Mineralocorticoid receptor antagonists (MRA) | 3 (2.9%) | 1 (2.3%) | 2 (3.4%) |  |
| Other anti-hypertensive drugs | 103 (100%) | 44 (100%) | 59 (100%) |  |
| Antiplatelet agents | 43 (42%) | 18 (41%) | 25 (42%) |  |
| Oral anticoagulants | 7 (6.8%) | 3 (6.8%) | 4 (6.8%) |  |
| Statins | 77 (75%) | 32 (73%) | 45 (76%) |  |
| Ezetimibe | 35 (34%) | 13 (30%) | 22 (37%) |  |
| Fibrates | 4 (3.9%) | 1 (2.3%) | 3 (5.1%) |  |
| Other anti-lipid medications | 12 (12%) | 6 (14%) | 6 (10%) |  |
| Other cardiovascular medications | 7 (6.8%) | 5 (11%) | 2 (3.4%) |  |
| **^1^**n(%). | | | | |

**Table S6.** Clinical and biohumoral data measured during the study from T1 (third month) to T4 (twelfth month) by allocated group and total group

|  |  | **T1** |  |  |  | **T2** |  |  |  | **T3** |  |  |  | **T4** |  |
| --- | --- | --- | --- | --- | --- | --- | --- | --- | --- | --- | --- | --- | --- | --- | --- |
| **Variables** | **Intervention**  **N = 51^1^** | **Control**  **N = 52^1^** | **p.value^2^** |  | **Intervention**  **N = 51^1^** | **Control**  **N =52^1^** | **p-value^2^** |  | **Intervention**  **N = 51^1^** | **Control**  **N = 52^1^** | **p-value^2^** |  | **Intervention,**  **N = 51^1^** | **Control,**  **N = 52^1^** | **p-value^2^** |
| HbA1c, mmol/mol | 54 (49, 61) | 56 (52, 61) | 0.04 |  | 54 (50, 60) | 56 (50, 65) | 0003 |  | 52 (51, 57) | 57 (51, 63) | 0.11 |  | 54 (49, 59) | 55 (50, 64) | 0.04 |
| Missing | 5 | 12 |  |  | 2 | 14 |  |  | 16 | 13 |  |  | 1 | 2 |  |
| Weight, Kg | 80 (74, 92) | 79 (69, 87) | 0.06 |  | 79 (71, 89) | 78 (67, 88) | 0007 |  | 80 (71, 91) | 79 (67, 86) | 0.03 |  | 80 (70, 90) | 80 (68, 87) | 0.06 |
| Missing | 2 | 6 |  |  | 6 | 7 |  |  | 7 | 4 |  |  | 2 | 3 |  |
| Cholesterol LDL, mg/dl | - | - |  |  | - | - |  |  | - | - |  |  | 50 (41, 59) | 46 (39, 58) | 0.04 |
| Missing |  |  |  |  |  |  |  |  |  |  |  |  | 1 | 5 |  |
| Cholesterol non-HDL, mg/dl | - | - |  |  | - | - |  |  | - | - |  |  | 81 (64, 98) | 79 (64, 101) | >0.9 |
| SPA, mmHg | 128 (121, 134) | 130 (120, 136) | 0.05 |  | 130 (120, 138) | 130 (120, 135) | >0.9 |  | 129 (122, 137) | 132 (120, 140) | 0.05 |  | 124 (118, 138) | 122 (110, 132) | 0.05 |
| Missing | 3 | 10 |  |  | 6 | 11 |  |  | 6 | 10 |  |  | 2 | 3 |  |
| DPA, mmHg | 75 (70, 79) | 78 (70, 81) | 0.02 |  | 77 (71, 81) | 78 (70, 80) | 0009 |  | 75 (71, 80) | 73 (70, 80) | 0.08 |  | 70 (70, 80) | 70 (70, 80) | 0.06 |
| Missing | 3 | 10 |  |  | 6 | 12 |  |  | 6 | 10 |  |  | 2 | 3 |  |
| IPAQ score | 2100 (949, 3628) | 1755 (525, 3596) | 0.07 |  | 788 (304, 1575) | 544 (163, 1073) | 0002 |  | 1050 (514, 1733) | 1050 (405, 1928) | >0.9 |  | 1935 (536, 4695) | 1425 (630, 3870) | 0.05 |
| Missing | 1 | 6 |  |  | 5 | 2 |  |  | 7 | 5 |  |  | 3 | 0 |  |
| N weight checks/last month | 4 (4, 5) | 4 (1, 9) | 0.04 |  | 4 (4, 4) | 4 (1, 5) | 0.07 |  | 4 (2, 4) | 4 (1, 4) | 0.04 |  | 4 (3, 5) | 4 (1, 9) | 0.03 |
| N BP checks/last month | 4.3 (4.0, 4.7) | 4.3 (1.0, 4.3) | 0.082 |  | 4.3 (4.3, 5.2) | 2.0 (1.0, 4.3) | 0.005 |  | 4 (3, 4) | 3 (1, 5) | 0.03 |  | 4 (4, 9) | 4 (2, 7) | 0.05 |
| N HbA1c checks/trimester | 1.00 (1.00, 1.00) | 1.00 (0.75, 1.00) | 0.10 |  | 1.00 (1.00, 1.00) | 1.00 (0.00, 1.00) | 0.01 |  | 1.00 (0.00, 1.00) | 1.00 (0.00, 1.00) | 0.09 |  | 1.00 (1.00, 1.00) | 1.00 (1.00, 1.00) | 0.08 |
| N contact with CAD/trimester | 0 (0, 0) | 0 (0, 0) | 0.05 |  | 0 (0, 0) | 0 (0, 1.00) | 0.03 |  | 1.00 (0, 1.00) | 0 (0, 1.00) | 0.06 |  | 0 (0, 0) | 0 (0, 0) | 0.03 |
| N hypoglycemic episodes | 0 (0, 0) | 0 (0, 0) | >0.9 |  | 0 (0, 0) | 0 (0, 0) | >0.9 |  | 0 (0, 0) | 0 (0, 0) | 0.02 |  | 0 (0, 0) | 0 (0, 0) | 0.02 |
| N cardiological visits | 0 (0, 0) | 0 (0, 0) | 0.03 |  | 0 (0, 0) | 0 (0, 0) | >0.9 |  | 0 (0, 0) | 0 (0, 0) | >0.9 |  | 0 (0, 0) | 0 (0, 0) | 0.02 |
| N cerebrovascular or nephrovascular visits | 0 (0, 0) | 0 (0, 0) |  |  | 0 (0, 0) | 0 (0, 0) |  |  | 0 (0, 0) | 0 (0, 0) | 0.06 |  | 0 (0, 0) | 0 (0, 0) | 0.03 |
| N hospitalisation for diabetes or cardiovascular complications | 0 (0, 0) | 0 (0, 0) |  |  | 0 (0, 0) | 0 (0, 0) | 0.03 |  | 0 (0, 0) | 0 (0, 0) | 0.06 |  | 0 (0, 0) | 0 (0, 0) | 0.03 |
| N change in treatment | 2.00 (1.00, 3.00) | 1.00 (0.75, 3.00) | 0.02 |  | 0 (0, 1.00) | 0 (0, 1.00) | 0.05 |  | 0 (0, 2.00) | 0.50 (0, 2.00) | 0.09 |  | 0 (0, 0) | 0 (0, 1.00) | 0.04 |
| ^1^Median (interquartile range); ^2^Wilcoxon rank sum test; **Abbreviations:** HDL = high-density lipoprotein; IQR = interquartile range; LDL = low-density lipoprotein; N = number; T1 = 3 months; T2 = 6 months; T3 = 9 months; T4 = 12 months. **Notes:** the number of blood pressure and weight checks refers to a period of one month (last month with respect to the follow-up time), while the number of visits/hospitalizations, changes in therapy, contacts with the diabetes center, episodes of hypoglycemia refer to the quarter preceding the follow-up time | | | | | | | | | | | | | | | |

**Table S7.** Exploratory post-hoc analysis on the key characteristics of application usage

| **Domain** | **Application usage characteristics** | **N = 51^1^** |  |
| --- | --- | --- | --- |
| A | Number of data registration for drug therapy intake | 45 (0, 1493) |  |
|  | N of expected medication entries | 2720 (1747, 3652) |  |
|  | Medication adherence (0-100)^2^ | 2 (0, 64) |  |
|  | Missing | 1 |  |
| B | N of total entries of clinical parameters | 206 (89, 312) |  |
|  | N of entries following clinical parameters reminders | 123 (21, 224) |  |
|  | N of expected clinical parameter to enter | 219 (156, 343) |  |
|  | Clinical parameters adherence (0-100)^3^ | 50 (20, 100) |  |
|  | Missing | 2 |  |
|  | Clinical parameters entries/ Clinical parameters expected (0-100) | 100 (50, 150) |  |
|  | Missing | 2 |  |
| C | N of messages written by participants | 6 (0, 17) |  |
|  | N of messages written by a clinician | 12 (0, 17) |  |
|  | N of messages by participant/N of messages by clinical | 0.75 (0.40, 1.41) |  |
|  | Missing | 14 |  |
| ^1^Median (IQR); ^2^Adherence to therapy was calculated from the ratio between registration of drug therapy intake and expected number of therapies to be included, multiplied by 100. Clinical parameters include blood sugar, blood pressure, heart rate and body weight; ^3^Adherence to clinical parameters was calculated from the ratio of number of entries following reminders of clinical parameters and expected number of clinical parameters to be entered, multiplied by 100. **Abbreviations**: IQR = interquartile range; N = number. | | | |
