# Supplementary material for: Impact of digital health on Type 2 diabetes management: a randomised controlled trial of the ‘TreC Diabete’ platform (TELEMECHRON Study)
Source: Front Clin Diabetes Healthc. 2025 Jun 10;6:1589548. doi: 10.3389/fcdhc.2025.1589548 (PMC12186056; doi:10.3389/fcdhc.2025.1589548)
Supplement: Supplementary file 2 [file DataSheet2.docx]

**SENSITIVITY ANALYSES**

**Summary**

[**Intention-to-treat approach (ITT approach)** 3](#_Toc190344595)

[*Scenario 1: no imputation was applied to the missing data* 3](#_Toc190344596)

[**Table SS1**. Baseline, 12-month, and changes (Δ) between the two time points in HbA1c levels, by intervention and control groups 3](#_Toc190344597)

[**Table SS2.** Change in clinical feature values between baseline and follow-up at 12 months. 3](#_Toc190344598)

[**Table SS3.** Percentage of patients who achieved the therapeutic target for the parameters of interest. 3](#_Toc190344599)

[**Table SS4.** Baseline, 12-month, and changes (Δ) between the two time points in HbA1c levels. 4](#_Toc190344600)

[*Scenario 2: missing data from the intervention group were imputed using the most extreme negative values observed in the entire sample, while the most extreme positive values were used for the control group* 5](#_Toc190344601)

[**Table SS5.** Baseline, 12-month, and changes (Δ) between the two time points in HbA1c levels, by intervention and control groups**.** 5](#_Toc190344602)

[**Table SS6.** Change in clinical feature values between baseline and follow-up at 12 months. 5](#_Toc190344603)

[**Table SS7.** Percentage of patients who achieved the therapeutic target for the parameters of interest. 5](#_Toc190344604)

[**Table SS8. Baseline, 12-month, and changes (Δ) between the two time points in HbA1c levels** 6](#_Toc190344605)

[*Scenario 3: missing data from the control group were imputed using the most extreme negative values observed in the entire sample, while the most extreme positive values were used for the intervention group* 7](#_Toc190344606)

[**Table SS9.** Baseline, 12-month, and changes (Δ) between the two time points in HbA1c levels, by intervention and control groups. 7](#_Toc190344607)

[**Table SS10.** Change in clinical feature values between baseline and follow-up at 12 months. 7](#_Toc190344608)

[**Table SS11.** Percentage of patients who achieved the therapeutic target for the parameters of interest. 7](#_Toc190344609)

[**Table SS12.** Baseline, 12-month, and changes (Δ) between the two time points in HbA1c levels. 8](#_Toc190344610)

[**Per-protocol (PP) approach** 9](#_Toc190344611)

[**Table SS13.** Baseline, 12-month, and changes (Δ) between the two time points in HbA1c levels, by intervention and control groups 9](#_Toc190344612)

[*Scenario 0: missing data were imputed using the multiple imputation by chained equations (MICE) algorithm* 9](#_Toc190344613)

[**Table SS14. C**hange in clinical feature values between baseline and follow-up at 12 months. 9](#_Toc190344614)

[**Table SS15.** Percentage of patients who achieved the therapeutic target for the parameters of interest. 9](#_Toc190344615)

[**Table SS16.** Baseline, 12-month, and changes (Δ) between the two time points in HbA1c levels. 10](#_Toc190344616)

[*Scenario 1: no imputation was applied to the missing data.* 11](#_Toc190344617)

[**Table SS17.** Change in clinical feature values between baseline and follow-up at 12 months. 11](#_Toc190344618)

[**Table SS18.** Percentage of patients who achieved the therapeutic target for the parameters of interest. 11](#_Toc190344619)

[**Table SS19.** Baseline, 12-month, and changes (Δ) between the two time points in HbA1c levels. 12](#_Toc190344620)

[*Scenario 2: missing data from the intervention group were imputed using the most extreme negative values observed in the entire sample, while the most extreme positive values were used for the control group* 13](#_Toc190344621)

[**Table SS20.** Change in clinical feature values between baseline and follow-up at 12 months 13](#_Toc190344622)

[**Table SS21.** Percentage of patients who achieved the therapeutic target for the parameters of interest. 13](#_Toc190344623)

[**Table SS22.** Baseline, 12-month, and changes (Δ) between the two time points in HbA1c levels. 14](#_Toc190344624)

[*Scenario 3: missing data from the control group were imputed using the most extreme negative values observed in the entire sample, while the most extreme positive values were used for the intervention group.* 15](#_Toc190344625)

[**Table SS23.** Change in clinical feature values between baseline and follow-up at 12 months. 15](#_Toc190344626)

[**Table SS24.** Percentage of patients who achieved the therapeutic target for the parameters of interest. 15](#_Toc190344627)

[**Table SS25.** Baseline, 12-month, and changes (Δ) between the two time points in HbA1c levels 16](#_Toc190344628)

[**As-trated (AT) approach** 17](#_Toc190344629)

[*Scenario 0: missing data were imputed using the multiple imputation by chained equations (MICE) algorithm* 17](#_Toc190344630)

[**Table SS26.** Baseline, 12-month, and changes (Δ) between the two time points in HbA1c levels, by intervention and control groups. 17](#_Toc190344631)

[**Table SS27.** Change in clinical feature values between baseline and follow-up at 12 months. 17](#_Toc190344632)

[**Table SS28.** Percentage of patients who achieved the therapeutic target for the parameters of interest. 17](#_Toc190344633)

[**Table SS29.** Baseline, 12-month, and changes (Δ) between the two time points in HbA1c levels. 18](#_Toc190344634)

[*Scenario 1: no imputation was applied to the missing data* 19](#_Toc190344635)

[**Table SS30.** Baseline, 12-month, and changes (Δ) between the two time points in HbA1c levels, by intervention and control groups 19](#_Toc190344636)

[**Table SS31.** Change in clinical feature values between baseline and follow-up at 12 months 19](#_Toc190344637)

[**Table SS32.** Percentage of patients who achieved the therapeutic target for the parameters of interest. 19](#_Toc190344638)

[**Table SS33.** Baseline, 12-month, and changes (Δ) between the two time points in HbA1c levels 20](#_Toc190344639)

[*Scenario 2: missing data from the intervention group were imputed using the most extreme negative values observed in the entire sample, while the most extreme positive values were used for the control group* 21](#_Toc190344640)

[**Table SS34**. Baseline, 12-month, and changes (Δ) between the two time points in HbA1c levels, by intervention and control groups 21](#_Toc190344641)

[**Table SS35.** Change in clinical feature values between baseline and follow-up at 12 months 21](#_Toc190344642)

[**Table SS36.** Percentage of patients who achieved the therapeutic target for the parameters of interest. 21](#_Toc190344643)

[**Table SS37.** Baseline, 12-month, and changes (Δ) between the two time points in HbA1c levels. 22](#_Toc190344644)

[*Scenario 3: missing data from the control group were imputed using the most extreme negative values observed in the entire sample, while the most extreme positive values were used for the intervention group.* 23](#_Toc190344645)

[**Table SS38.** Baseline, 12-month, and changes (Δ) between the two time points in HbA1c levels, by intervention and control groups. 23](#_Toc190344646)

[**Table SS39.** Change in clinical feature values between baseline and follow-up at 12 months. 23](#_Toc190344647)

[**Table SS40.** Percentage of patients who achieved the therapeutic target for the parameters of interest. 23](#_Toc190344648)

[**Table SS41.** Baseline, 12-month, and changes (Δ) between the two time points in HbA1c levels. 24](#_Toc190344649)

# **Intention-to-treat approach (ITT approach)**

## *Scenario 1: no imputation was applied to the missing data*

### **Table SS1**. Baseline, 12-month, and changes (Δ) between the two time points in HbA1c levels, by intervention and control groups

|  | Baseline^1^ | T4^1^ | p-value^2^ | ∆ (T4- Baseline)^1^ | p-value^3^ | |
| --- | --- | --- | --- | --- | --- | --- |
| HbA1c, mmol/mol |  |  |  |  | >0.9 | |
| Intervention | 60 (57, 68) | 54 (49, 59) | <0.001 | -6 (-11, -3) |  | |
| Control | 63 (59, 70) | 55 (50, 64) | <0.001 | -7 (-14, -1) |  | |
| ^1^Median (interquartile range); ^2^Wilcoxon sign-ranked test comparing HbA1c at baseline and at T4; ^3^Wilcoxon rank sum test comparing haemoglobin glycated changes between the two groups; **Abbreviations**: HbA1c = glycated haemoglobin. | | | | | |  |

### **Table SS2.** Change in clinical feature values between baseline and follow-up at 12 months.

|  | Intervention^1^  N = 51 | Control^1^  N = 52 | p-value^2^ |  |
| --- | --- | --- | --- | --- |
| ∆Weight, Kg | -2.20 (-4.9, 0.4) | -3.0 (-4.8, -0.9) | 0.5 |  |
| ∆Cholesterol LDL, mg/dl | -23 (-56, -3) | -20 (-62, 2) | 0.8 |  |
| ∆Cholesterol non-HDL, mg/dl | -7 (-35, 2) | -12 (-29, 5) | 0.9 |  |
| ∆sBP, mmHg | -1 (-10, 10) | -5 (-16, 6) | 0.2 |  |
| ∆dBP, mmHg | 0 (-5, 6) | 0 (-8, 8) | 0.8 |  |
| ^1^Median (interquartile range); ^2^Wilcoxon rank-sum test; **Abbreviations**: ∆ = Difference between baseline and follow-up at 12 months;  Kg= kilograms; LDL = low-density lipoprotein; HDL = high-density lipoprotein; sBP = systolic blood pressure; dBP = diastolic blood pressure. | | | | |

### **Table SS3.** Percentage of patients who achieved the therapeutic target for the parameters of interest.

|  |  | Baseline |  |  |  | T4 |  |
| --- | --- | --- | --- | --- | --- | --- | --- |
| Variables | Intervention  N = 51^1^ | Control  N = 52^1^ | p-value^2^ |  | Intervention  N = 51^1^ | Control  N = 52^1^ | p-value^2^ |
| HbA1c target | 0 (0%) | 0 (0%) | - |  | 26 (51%) | 22 (42%) | 0.4 |
| Hypoglicemic episodes | - | - | - |  | 1 (2.0%) | 4 (7.7%) | 0.4 |
| Lipid target | 23 (45%) | 25 (48%) | 0.8 |  | 36 (71%) | 33 (63%) | 0.4 |
| BP target | 36 (71%) | 37 (71%) | >0.9 |  | 38 (75%) | 38 (73%) | 0.9 |
| HbA1c +lipid+ BPcombined target | - | - | - |  | 13 (25%) | 10 (19%) | 0.4 |
| ^1^number (%); ^2^Pearson's Chi-squared test; Fisher's exact test; **Abbreviations:** HbA1c = haemoglobin glycated; BP = blood pressure; T4 = 12 months. **Notes:** hypoglycemia is defined as a blood glucose level below 70 mg/dl or symptoms suggestive of it (e.g.shaking, sweating, dizziness); no baseline participants had a target HbA1c level (according to eligibility criteria). | | | | | | | |

### **Table SS4.** Baseline, 12-month, and changes (Δ) between the two time points in HbA1c levels.

|  | Baseline | | |  | T4 | | |  | ∆(T4-Baseline) | | | |  |
| --- | --- | --- | --- | --- | --- | --- | --- | --- | --- | --- | --- | --- | --- |
| Questionnaires | Intervention^1^ | Control^1^ | p-value^2^ |  | Intervention^1^ | Control^1^ | p-value^2^ |  | Intervention^1^ | Control^1^ | p-value^2^ | | |
|  | N = 51 | N = 52 |  |  | N = 51 | N = 52 |  |  | N = 51 | N = 52 |  | | |
| SF – PCS-12 | 50 (42, 54) | 51 (42, 54) | 0.7 |  | 51 ( 44, 54) | 49 (42, 54) | 0.7 |  | 0 (-2, 5) | 0 (-5, 3) | 0.5 | | |
| SF – MCS-12 | 54 (46, 59) | 51 (45, 56) | 0.04 |  | 55 (48, 59) | 51 (46, 56) | 0.08 |  | -4 (-8, 1) | -2 (-6, 6) | 0.5 | | |
| MMAS-8 | 8.00 (6.00, 8.00) | 7.75 ( 6.75, 8.00) | 0.5 |  | 8.00 (6.63, 8.00) | 8.00 (7.00,  8.00) | 0.5 |  | 0.00 (0.00, 0.25) | 0.00 (0.00, 0.75) | 0.5 | | |
| IPAQ (Mets) | 3946 (1223, 7200) | 1548 (380, 3248) | <0.001 |  | 1935 (536-4695) | 1740 (670,  4050) | 0.8 |  | -375 (-2980, 891) | 475 (-488, 1256) | 0.03 | | |
| DASI (score) | 50 (31, 58) | 46 (35, 58) | 0.8 |  | 45  (32, 58) | 48 (29, 58) | 0.8 |  | 0 (-5, 1) | 0 (-8, 2) | >0.9 | | |
| DASI (Mets) | 8.91 (6.61, 9.89) | 8.42 (7.01, 9.89) | 0.7 |  | 8.23 (6.68, 9.89) | 8.61 (6.36, 9.89) | 0.8 |  | 0.00 (-0.57, 0.07) | 0.00 (-0.99, 0.28) | 0.9 | | |
| ^1^Median (interquartile range); ^2^Wilcoxon rank-sum test; **Abbreviations**: T4 = 12 months ; SF – PCS-12 = Short Form (Quality of life questionnaire) physical component score SF-PCS- 12; SF – MCS-12 = Short Form (Quality of life questionnaire) mental component score – 12; MMAS-8 = Morisky Medication Adherence Scale in the 8-item version; IPAQ = Physical Activity Questionnaire Daily; DASI = Duke Activity Status Index. | | | | | | | | | | | |  |  |

## *Scenario 2: missing data from the intervention group were imputed using the most extreme negative values observed in the entire sample, while the most extreme positive values were used for the control group*

### **Table SS5.** Baseline, 12-month, and changes (Δ) between the two time points in HbA1c levels, by intervention and control groups**.**

|  | Baseline^1^ | T4^1^ | p-value^2^ | ∆ (T4- Baseline)^1^ | p-value^3^ |
| --- | --- | --- | --- | --- | --- |
| HbA1c, mmol/mol |  |  |  |  | 0.6 |
| Intervention | 60 (57, 68) | 54 (49, 60) | <0.001 | -6 (-11, -3) |  |
| Control | 63 (59, 70) | 54 (50, 63) | <0.001 | -8 (-15, -2) |  |
| ^1^Median (interquartile range); ^22^Wilcoxon sign-ranked test comparing HbA1c at baseline and at T4; ^3^Wilcoxon rank sum test comparing haemoglobin glycated changes between the two groups; **Abbreviations**: HbA1c = glycated haemoglobin. | | | | | |

### **Table SS6.** Change in clinical feature values between baseline and follow-up at 12 months.

|  | Intervention^1^  N = 51 | Control^1^  N = 52 | p-value^2^ |  |
| --- | --- | --- | --- | --- |
| ∆Weight, Kg | -2.1 (-4.9, 0.6) | -3.5 (-6.1, -1.0) | 0.14 |  |
| ∆Cholesterol LDL, mg/dl | -23 (-55, -1) | -28 (-85, 1) | 0.2 |  |
| ∆Cholesterol non-HDL, mg/dl | -6 (-35, 3) | -15 (-48, 2) | 0.2 |  |
| ∆sBP, mmHg | 0 (-10, 12) | -6 (-17, 5) | 0.05 |  |
| ∆dBP, mmHg | 0 (-5, 6) | 0 (-10, 7) | 0.3 |  |
| ^1^Median (interquartile range); ^2^Wilcoxon rank-sum test; **Abbreviations**: ∆ = Difference between baseline and follow-up at 12 months;  Kg= kilograms; LDL = low-density lipoprotein; HDL = high-density lipoprotein; sBP = systolic blood pressure; dBP = diastolic blood pressure. | | | | |

### **Table SS7.** Percentage of patients who achieved the therapeutic target for the parameters of interest.

|  |  | Baseline |  |  |  | T4 |  |
| --- | --- | --- | --- | --- | --- | --- | --- |
| Variables | Intervention  N = 51^1^ | Control  N = 52^1^ | p-value^2^ |  | Intervention  N = 51^1^ | Control  N = 52^1^ | p-value^2^ |
| HbA1c target | 0 (0%) | 0 (0%) | - |  | 25 (49%) | 23 (44%) | 0.6 |
| Hypoglicemic episodes | - | - | - |  | 1 (2.0%) | 4 (7.7%) | 0.4 |
| Lipid target | 23 (45%) | 25 (48%) | 0.8 |  | 35 (69%) | 32 (62%) | 0.5 |
| BP target | 36 (71%) | 37 (71%) | >0.9 |  | 37 (73%) | 39 (75%) | 0.8 |
| HbA1c +lipid+ BPcombined target | - | - | - |  | 13 (25%) | 12 (23%) | 0.8 |
| ^1^number (%); ^2^Pearson's Chi-squared test; Fisher's exact test; **Abbreviations:** HbA1c = haemoglobin glycated; BP = blood pressure; T4 = 12 months. **Notes:** hypoglycemia is defined as a blood glucose level below 70 mg/dl or symptoms suggestive of it (e.g.shaking, sweating, dizziness); no baseline participants had a target HbA1c level (according to eligibility criteria). | | | | | | | |

### **Table SS8. Baseline, 12-month, and changes (Δ) between the two time points in HbA1c levels**

|  | Baseline | | |  | T4 | | |  | ∆(T4-Baseline) | | | |
| --- | --- | --- | --- | --- | --- | --- | --- | --- | --- | --- | --- | --- |
| Questionnaires | Intervention^1^ | Control^1^ | p-value^2^ |  | Intervention^1^ | Control^1^ | p-value^2^ |  | Intervention^1^ | Control^1^ | p-value^2^ | |
|  | N = 51 | N = 52 |  |  | N = 51 | N = 52 |  |  | N = 51 | N = 52 |  | |
| SF – PCS-12 | 50 (42, 54) | 51 (42, 54) | 0.7 |  | 50 (41, 54) | 50 (43, 55) | 0.6 |  | 0 (-4, 5) | 1 (-4, 4) | 0.8 | |
| SF – MCS-12 | 54 (46, 59) | 51 (45, 56) | 0.042 |  | 54 (47, 58) | 53 (46, 58) | 0.5 |  | -4 (-9, 1) | -1 (-6, 6) | 0.2 | |
| MMAS-8 | 8.00 (6.00, 8.00) | 7.75 (6.75, 8.00) | 0.8 |  | 7.75 (6.25, 8.00) | 8.00 (7.00, 8.00) | 0.11 |  | 0.00 (-0.25, 0.25) | 0.00 (0.00, 0.31) | 0.4 | |
| IPAQ (Mets) | 3810 (895, 7080) | 1548 (380, 3248) | 0.002 |  | 1890 (338, 4545) | 1980 (690, 4888) | 0.6 |  | -470 (-3361, 483) | 621 (-473, 1474) | 0.008 | |
| DASI (score) | 50 (31, 58) | 46 (35, 58) | 0.8 |  | 45 (29, 58) | 49 (31, 58) | 0.4 |  | 0 (-6, 0) | 0 (-8, 3) | 0.6 | |
| DASI (Mets) | 8.91 (6.61, 9.89) | 8.42 (7.01, 9.89) | 0.7 |  | 8.23 (6.27, 9.89) | 8.81 (6.53, 9.89) | 0.4 |  | 0.00 (-0.71, 0.00) | 0.00 (-0.98, 0.39) | 0.6 | |
| ^1^Median (interquartile range); ^2^Wilcoxon rank-sum test; **Abbreviations**: T4 = 12 months ; SF – PCS-12 = Short Form (Quality of life questionnaire) physical component score SF-PCS-12; SF – MCS-12 = Short Form (Quality of life questionnaire) mental component score – 12; MMAS-8 = Morisky Medication Adherence Scale in the 8-item version; IPAQ = Physical Activity Questionnaire Daily; DASI = Duke Activity Status Index. | | | | | | | | | | | |  |

## *Scenario 3: missing data from the control group were imputed using the most extreme negative values observed in the entire sample, while the most extreme positive values were used for the intervention group*

### **Table SS9.** Baseline, 12-month, and changes (Δ) between the two time points in HbA1c levels, by intervention and control groups.

|  | Baseline^1^ | T4^1^ | p-value^2^ | ∆ (T4- Baseline)^1^ | p-value^3^ |
| --- | --- | --- | --- | --- | --- |
| HbA1c, mmol/mol |  |  |  |  | 0.7 |
| Intervention | 60 (57, 68) | 53 (49, 59) | <0.001 | -6 (-12, -4) |  |
| Control | 63 (59, 70) | 55 (50, 65) | <0.001 | -7 (-13, 0) |  |
| ^1^Median (interquartile range); ^2^Wilcoxon sign-ranked test comparing HbA1c at baseline and at T4; ^3^Wilcoxon rank sum test comparing haemoglobin glycated changes between the two groups; **Abbreviations**: HbA1c = glycated haemoglobin. | | | | | |

### **Table SS10.** Change in clinical feature values between baseline and follow-up at 12 months.

|  | Intervention^1^  N = 51 | Control^1^  N = 52 | p-value^2^ |  |
| --- | --- | --- | --- | --- |
| ∆Weight, Kg | -2.6 (-5.0, 0.2) | -3.0 (-4.6, 0.0) | 0.9 |  |
| ∆Cholesterol LDL, mg/dl | -23 (-57, -4) | -18 (-55, 9) | 0.4 |  |
| ∆Cholesterol non-HDL, mg/dl | -8 (-36, 2) | -9 (-28, 11) | 0.3 |  |
| ∆sBP, mmHg | -4 (-10, 8) | -4 (-13, 10) | 0.8 |  |
| ∆dBP, mmHg | 0 (-6, 5) | 0 (-8, 10) | 0.5 |  |
| ^1^Median (interquartile range); ^2^Wilcoxon rank-sum test; **Abbreviations**: ∆ = Difference between baseline and follow-up at 12 months;  Kg= kilograms; LDL = low-density lipoprotein; HDL = high-density lipoprotein; sBP = systolic blood pressure; dBP = diastolic blood pressure. | | | | |

### **Table SS11.** Percentage of patients who achieved the therapeutic target for the parameters of interest.

|  |  | Baseline |  |  |  | T4 |  |
| --- | --- | --- | --- | --- | --- | --- | --- |
| Variables | Intervention  N = 51^1^ | Control  N = 52^1^ | p-value^2^ |  | Intervention  N = 51^1^ | Control  N = 52^1^ | p-value^2^ |
| HbA1c target | 0 (0%) | 0 (0%) | - |  | 26 (51%) | 21 (40%) | 0.3 |
| Hypoglicemic episodes | - | - | - |  | 1 (2.0%) | 4 (7.7%) | 0.4 |
| Lipid target | 23 (45%) | 24 (46%) | >0.9 |  | 36 (71%) | 29 (56%) | 0.12 |
| BP target | 36 (71%) | 37 (71%) | >0.9 |  | 39 (76%) | 36 (69%) | 0.4 |
| HbA1c +lipid+ BPcombined target | - | - | - |  | 14 (27%) | 9 (17%) | 0.2 |
| ^1^number (%); ^2^Pearson's Chi-squared test; Fisher's exact test; **Abbreviations:** HbA1c = haemoglobin glycated; BP = blood pressure; T4 = 12 months. **Notes:** hypoglycemia is defined as a blood glucose level below 70 mg/dl or symptoms suggestive of it (e.g.shaking, sweating, dizziness); no baseline participants had a target HbA1c level (according to eligibility criteria). | | | | | | | |

### **Table SS12.** Baseline, 12-month, and changes (Δ) between the two time points in HbA1c levels.

|  | Baseline | | |  | T4 | | |  | ∆(T4-Baseline) | | | |
| --- | --- | --- | --- | --- | --- | --- | --- | --- | --- | --- | --- | --- |
| Questionnaires | Intervention^1^ | Control^1^ | p-value^2^ |  | Intervention^1^ | Control^1^ | p-value^2^ |  | Intervention^1^ | Control^1^ | p-value^2^ | |
|  | N = 51 | N = 52 |  |  | N = 51 | N = 52 |  |  | N = 51 | N = 52 |  | |
| SF – PCS-12 | 50 (42, 54) | 51 (42, 54) | 0.7 |  | 52 (45, 54) | 49 (41, 54) | 0.2 |  | 1 (-2, 6) | 0 (-5, 3) | 0.13 | |
| SF – MCS-12 | 54 (46, 59) | 51 (45, 56) | 0.042 |  | 56 (49, 59) | 51 (44, 56) | 0.010 |  | -4 (-8, 2) | -4 (-7, 4) | >0.9 | |
| MMAS-8 | 8.00 (6.00, 8.00) | 7.00 (6.00, 8.00) | 0.2 |  | 8.00 (6.75, 8.00) | 7.88 (6.94, 8.00) | 0.8 |  | 0.00 (0.00, 0.25) | 0.00 (0.00, 1.00) | 0.4 | |
| IPAQ (Mets) | 4080 (1395, 8220) | 1548 (380, 3248) | <0.001 |  | 2133 (585, 5450) | 1425 (630, 3870) | 0.3 |  | -225 (-2980, 1515) | 283 (-861, 1249) | 0.14 | |
| DASI (score) | 50 (31, 58) | 46 (35, 58) | 0.8 |  | 45 (34, 58) | 46 (26, 58) | 0.6 |  | 0 (-5, 1) | 0 (-10, 1) | 0.5 | |
| DASI (Mets) | 8.91 (6.61, 9.89) | 8.42 (7.01, 9.89) | 0.7 |  | 8.33 (6.87, 9.89) | 8.42 (5.97, 9.89) | 0.6 |  | 0.00 (-0.55, 0.14) | 0.00 (-1.18, 0.16) | 0.5 | |
| ^1^Median (interquartile range); ^2^Wilcoxon rank-sum test; **Abbreviations**: T4 = 12 months ; SF – PCS-12 = Short Form (Quality of life questionnaire) physical component score SF-PCS-12; SF – MCS-12 = Short Form (Quality of life questionnaire) mental component score – 12; MMAS-8 = Morisky Medication Adherence Scale in the 8-item version; IPAQ = Physical Activity Questionnaire Daily; DASI = Duke Activity Status Index. | | | | | | | | | | | |  |

# **Per-protocol (PP) approach**

For the primary outcome — changes (Δ) between the baseline ad 12-months in glycated haemoglobin (HbA1c) — non imputation was performed because patients with missing HbA1c information were excluded. So only one scenario was considered.

### **Table SS13.** Baseline, 12-month, and changes (Δ) between the two time points in HbA1c levels, by intervention and control groups

|  | **Baseline^1^** | **T4^1^** | **p-value^2^** | **∆ (T4- Baseline)^1^** | **p-value^3^** |
| --- | --- | --- | --- | --- | --- |
| HbA1c, mmol/mol |  |  |  |  | 0.7 |
| Intervention | 59 (56, 66) | 54 (49, 58) | <0.001 | -6 (-11, 3) |  |
| Control | 63 (59, 71) | 55 (50, 64) | <0.001 | -7 (-14, -1) |  |
| ^1^Median (interquartile range); ^2^Wilcoxon sign-ranked test comparing HbA1c at baseline and at T4; ^3^Wilcoxon rank sum test comparing haemoglobin glycated changes between the two groups; **Abbreviations**: HbA1c = glycated haemoglobin. | | | | | |

## *Scenario 0: missing data were imputed using the multiple imputation by chained equations (MICE) algorithm*

### **Table SS14. C**hange in clinical feature values between baseline and follow-up at 12 months.

| **Variables** | **Intervention^1^**  **N = 44** | **Control^1^**  **N = 50** | **p-value^2^** |
| --- | --- | --- | --- |
| ∆Weight, Kg | -2.4 (-4.9, 0.4) | -3.2 (-5.6, -0.9) | 0.4 |
| ∆Cholesterol LDL, mg/dl | -17 (-48, -2) | -20 (-60, 1) | 0.5 |
| ∆Cholesterol non-HDL, mg/dl | -10 (-35, 4) | -12 (-32, 7) | >0.9 |
| ∆sBP, mmHg | -4 (-10, 7) | -5 (-15, 7) | 0.4 |
| ∆dBP, mmHg | 0 (-5, 6) | 0 (-8, 10) | >0.9 |
| ^1^Median (interquartile range); ^2^Wilcoxon rank-sum test; **Abbreviations**: ∆ = Difference between baseline and follow-up at 12 months;  Kg= kilograms; LDL = low-density lipoprotein; HDL = high-density lipoprotein; sBP = systolic blood pressure; dBP = diastolic blood pressure. | | | |

### **Table SS15.** Percentage of patients who achieved the therapeutic target for the parameters of interest.

|  |  | **Baseline** |  |  |  | **T4** |  |
| --- | --- | --- | --- | --- | --- | --- | --- |
| **Variables** | **Intervention**  **N = 44^1^** | **Control**  **N = 50^1^** | **p-value^2^** |  | **Intervention**  **N = 44^1^** | **Control**  **N = 50^1^** | **p-value^2^** |
| HbA1c target | 0 (0%) | 0 (0%) | - |  | 22 (50%) | 21 (42%) | 0.4 |
| Hypoglicemic episodes | - | - | - |  | 1 (2.3%) | 4 (8.0%) | 0.4 |
| Lipid target | 22 (50%) | 23 (46%) | 0.7 |  | 33 (75%) | 32 (64%) | 0.2 |
| BP target | 32 (73%) | 37 (74%) | 0.9 |  | 35 (80%) | 37 (74%) | 0.5 |
| HbA1c +lipid+ BP combined target | - | - | - |  | 13 (30%) | 10 (20%) | 0.3 |
| ^1^number (%); ^2^Pearson's Chi-squared test; Fisher's exact test; **Abbreviations:** HbA1c = haemoglobin glycated; BP = blood pressure; T4 = 12 months. **Notes:** hypoglycemia is defined as a blood glucose level below 70 mg/dl or symptoms suggestive of it (e.g.shaking, sweating, dizziness); no baseline participants had a target HbA1c level (according to eligibility criteria). | | | | | | | |

### **Table SS16.** Baseline, 12-month, and changes (Δ) between the two time points in HbA1c levels.

|  | Baseline | | |  | T4 | | |  | ∆(T4-Baseline) | | | |
| --- | --- | --- | --- | --- | --- | --- | --- | --- | --- | --- | --- | --- |
| Questionnaires | Intervention^1^ | Control^1^ | p-value^2^ |  | Intervention^1^ | Control^1^ | p-value^2^ |  | Intervention^1^ | Control^1^ | p-value^2^ | |
|  | N = 44 | N = 50 |  |  | N = 44 | N = 50 |  |  | N = 44 | N = 50 |  | |
| SF – PCS-12 | 50 (44, 54) | 51 (42, 55) | 0.7 |  | 52 (40, 54) | 49 (44, 54) | 0.8 |  | 0 (-2, 5) | 0 (-4, 4) | 0.6 | |
| SF – MCS-12 | 54 (45, 58) | 51 (44, 56) | 0.084 |  | 54 (48, 58) | 53 (46, 58) | 0.2 |  | -3 (-7, 3) | -3 (-6, 5) | 0.8 | |
| MMAS-8 | 8.00 (6.56, 8.00) | 7.38 (6.56, 8.00) | 0.3 |  | 8.00 (6.75, 8.00) | 8.00 (7.00, 8.00) | 0.9 |  | 0.00 (-0.06, 0.44) | 0.00 (0.00, 1.00) | 0.3 | |
| IPAQ (Mets) | 3655 (724, 6578) | 1548 (310, 3113) | 0.004 |  | 2160 (700, 5140) | 1980 (660, 4140) | 0.4 |  | -210 (-2078, 1478) | 475 (-488, 1256) | 0.2 | |
| DASI (score) | 51 (31, 58) | 46 (35, 58) | >0.9 |  | 45 (29, 58) | 48 (31, 58) | 0.4 |  | 0 (-5, 0) | 0 (-8, 2) | 0.6 | |
| DASI (Mets) | 8.97 (6.61, 9.89) | 8.42 (7.01, 9.89) | >0.9 |  | 8.23 (6.27, 9.89) | 8.70 (6.61, 9.89) | 0.4 |  | 0.00 (-0.65, 0.00) | 0.00 (-0.99, 0.28) | 0.6 | |
| ^1^Median (interquartile range); ^2^Wilcoxon rank-sum test; **Abbreviations**: T4 = 12 months ; SF – PCS-12 = Short Form (Quality of life questionnaire) physical component score SF-PCS-12; SF – MCS-12 = Short Form (Quality of life questionnaire) mental component score – 12; MMAS-8 = Morisky Medication Adherence Scale in the 8-item version; IPAQ = Physical Activity Questionnaire Daily; DASI = Duke Activity Status Index. | | | | | | | | | | | |  |

## *Scenario 1: no imputation was applied to the missing data.*

### **Table SS17.** Change in clinical feature values between baseline and follow-up at 12 months.

|  | Intervention^1^  N = 44 | Control^1^  N = 50 | p-value^2^ |  |
| --- | --- | --- | --- | --- |
| ∆Weight, Kg | -2.1 (-4.9, 0.5) | -3.0 (-4.8, -0.9) | 0.4 |  |
| ∆Cholesterol LDL, mg/dl | -17 (-48, -2) | -20 (-62, 2) | 0.5 |  |
| ∆Cholesterol non-HDL, mg/dl | -10 (-35, 4) | -12 (-29, 5) | >0.9 |  |
| ∆sBP, mmHg | -4 (-10, 8) | -5 (-16, 6) | 0.3 |  |
| ∆dBP, mmHg | 0 (-5, 6) | 0 (-8, 8) | 0.8 |  |
| ^1^Median (interquartile range); ^2^Wilcoxon rank-sum test; **Abbreviations**: ∆ = Difference between baseline and follow-up at 12 months;  Kg= kilograms; LDL = low-density lipoprotein; HDL = high-density lipoprotein; sBP = systolic blood pressure; dBP = diastolic blood pressure. | | | | |

### **Table SS18.** Percentage of patients who achieved the therapeutic target for the parameters of interest.

|  |  | Baseline |  |  |  | T4 |  |
| --- | --- | --- | --- | --- | --- | --- | --- |
| Variables | Intervention  N = 44^1^ | Control  N = 50^1^ | p-value^2^ |  | Intervention  N = 44^1^ | Control  N = 50^1^ | p-value^2^ |
| HbA1c target | 0 (0%) | 0 (0%) | - |  | 22 (50%) | 21 (42%) | 0.4 |
| Hypoglicemic episodes | - | - | - |  | 1 (2.3%) | 4 (8.0%) | 0.4 |
| Lipid target | 22 (50%) | 22 (45%) | 0.6 |  | 33 (75%) | 29 (59%) | 0.11 |
| BP target | 32 (73%) | 37 (74%) | 0.9 |  | 34 (79%) | 36 (73%) | 0.5 |
| HbA1c +lipid+ BP combined target | - | - | - |  | 13 (30%) | 9 (18%) | 0.2 |
| ^1^number (%); ^2^Pearson's Chi-squared test; Fisher's exact test; **Abbreviations:** HbA1c = haemoglobin glycated; BP = blood pressure; T4 = 12 months. **Notes:** hypoglycemia is defined as a blood glucose level below 70 mg/dl or symptoms suggestive of it (e.g.shaking, sweating, dizziness); no baseline participants had a target HbA1c level (according to eligibility criteria). | | | | | | | |

### **Table SS19.** Baseline, 12-month, and changes (Δ) between the two time points in HbA1c levels.

|  | **Baseline** | | |  | **T4** | | |  | | **∆(T4-Baseline)** | | | | |  |  |
| --- | --- | --- | --- | --- | --- | --- | --- | --- | --- | --- | --- | --- | --- | --- | --- | --- |
| **Questionnaires** | **Intervention^1^** | **Control^1^** | **p-value^2^** |  | **Intervention^1^** | **Control^1^** | **p-value^2^** | |  | | **Intervention^1^** | **Control^1^** | **p-value^2^** | | | |
|  | **N = 44** | **N = 50** |  |  | **N = 44** | **N = 50** |  | |  | | **N = 44** | **N = 50** |  | | | |
| SF – PCS-12 | 50 (44, 54) | 51 (42, 55) | 0.7 |  | 52 (42, 54) | 49 (42, 54) | 0.5 | |  | | 0 (-2, 5) | 0 (-5, 3) | 0.4 | | | |
| SF – MCS-12 | 54 (45, 58) | 51 (44, 56) | 0.08 |  | 55 (49, 59) | 52 (46, 56) | 0.08 | |  | | -4 (-8, 2) | -2 (-6, 6) | 0.6 | | | |
| MMAS-8 | 8.00 (6.56, 8.00) | 7.75 (6.75, 8.00) | 0.4 |  | 8.00 (6.75, 8.00) | 8.00 (7.00, 8.00) | 0.6 | |  | | 0.00 (-0.25, 0.25) | 0.00 (0.00, 0.75) | 0.3 | | | |
| IPAQ (Mets) | 3810 (895, 6646) | 1548 (310, 3113) | 0.003 |  | 2147 (639, 5040) | 1740 (670, 4050) | 0.4 | |  | | -195 (-2100, 1440) | 475 (-488, 1256) | 0.2 | | | |
| DASI (score) | 51 (31, 58) | 46 (35, 58) | >0.9 |  | 45 (30, 58) | 48 (29, 58) | 0.8 | |  | | 0 (-5, 0) | 0 (-8, 2) | 0.7 | | | |
| DASI (Mets) | 8.97 (6.61, 9.89) | 8.42 (7.01, 9.89) | >0.9 |  | 8.28 (6.38, 9.89) | 8.61 (6.36, 9.89) | 0.8 | |  | |  |  |  | | |  |
| ^1^Median (interquartile range); ^2^Wilcoxon rank-sum test; **Abbreviations**: T4 = 12 months ; SF – PCS-12 = Short Form (Quality of life questionnaire) physical component score SF-PCS-12; SF – MCS-12 = Short Form (Quality of life questionnaire) mental component score – 12; MMAS-8 = Morisky Medication Adherence Scale in the 8-item version; IPAQ = Physical Activity Questionnaire Daily; DASI = Duke Activity Status Index. | | | | | | | | | | | | | |  |  |  |

## *Scenario 2: missing data from the intervention group were imputed using the most extreme negative values observed in the entire sample, while the most extreme positive values were used for the control group*

### **Table SS20.** Change in clinical feature values between baseline and follow-up at 12 months

| **Variables** | **Intervention^1^**  **N = 44** | **Control^1^**  **N = 50** | **p-value^2^** |
| --- | --- | --- | --- |
| ∆Weight, Kg | -2.1 (-4.8, 0.5) | -3.2 (-5.6, -0.9) | 0.2 |
| ∆Cholesterol LDL, mg/dl | -17 (-48, -2) | -24 (-73, 1) | 0.2 |
| ∆Cholesterol non-HDL, mg/dl | -10 (-35, 4) | -15 (-42, 3) | 0.5 |
| ∆sBP, mmHg | -3 (-10, 11) | -6 (-16, 6) | 0.2 |
| ∆dBP, mmHg | 0 (-5, 6) | 0 (-10, 8) | 0.6 |
| ^1^Median (interquartile range); ^2^Wilcoxon rank-sum test; **Abbreviations**: ∆ = Difference between baseline and follow-up at 12 months;  Kg= kilograms; LDL = low-density lipoprotein; HDL = high-density lipoprotein; sBP = systolic blood pressure; dBP = diastolic blood pressure. | | | |

### **Table SS21.** Percentage of patients who achieved the therapeutic target for the parameters of interest.

|  |  | Baseline |  |  |  | T4 |  |
| --- | --- | --- | --- | --- | --- | --- | --- |
| Variables | Intervention  N = 44^1^ | Control  N = 50^1^ | p-value^2^ |  | Intervention  N = 44^1^ | Control  N = 50^1^ | p-value^2^ |
| HbA1c target | 0 (0%) | 0 (0%) | - |  | 22 (50%) | 21 (42%) | 0.4 |
| Hypoglicemic episodes | - | - | - |  | 1 (2.3%) | 4 (8.0%) | 0.4 |
| Lipid target | 22 (50%) | 23 (46%) | 0.7 |  | 33 (75%) | 30 (60%) | 0.12 |
| BP target | 32 (73%) | 37 (74%) | 0.9 |  | 34 (77%) | 37 (74%) | 0.7 |
| HbA1c +lipid+ BP combined target | - | - | - |  | 13 (30%) | 10 (20%) | 0.3 |
| ^1^number (%); ^2^Pearson's Chi-squared test; Fisher's exact test; **Abbreviations:** HbA1c = haemoglobin glycated; BP = blood pressure; T4 = 12 months. **Notes:** hypoglycemia is defined as a blood glucose level below 70 mg/dl or symptoms suggestive of it (e.g.shaking, sweating, dizziness); no baseline participants had a target HbA1c level (according to eligibility criteria). | | | | | | | |

### **Table SS22.** Baseline, 12-month, and changes (Δ) between the two time points in HbA1c levels.

|  | **Baseline** | | |  | **T4** | | |  | | **∆(T4-Baseline)** | | | | |  |
| --- | --- | --- | --- | --- | --- | --- | --- | --- | --- | --- | --- | --- | --- | --- | --- |
| **Questionnaires** | **Intervention^1^** | **Control^1^** | **p-value^2^** |  | **Intervention^1^** | **Control^1^** | **p-value^2^** | |  | | **Intervention^1^** | **Control^1^** | **p-value^2^** | | |
|  | **N = 44** | **N = 50** |  |  | **N = 44** | **N = 50** |  | |  | | **N = 44** | **N = 50** |  | | |
| SF – PCS-12 | 50 (44, 54) | 51 (42, 55) | 0.7 |  | 51 (40, 54) | 50 (42, 55) | 0.9 | |  | | 0 (-2, 5) | 0 (-4, 4) | 0.8 | | |
| SF – MCS-12 | 54 (45, 58) | 51 (44, 56) | 0.084 |  | 54 (48, 58) | 52 (46, 57) | 0.3 | |  | | -4 (-8, 1) | -2 (-6, 5) | 0.4 | | |
| MMAS-8 | 8.00 (6.56, 8.00) | 7.75 (6.75, 8.00) | 0.5 |  | 7.75 (6.50, 8.00) | 8.00 (7.00, 8.00) | 0.2 | |  | | 0.00 (-0.31, 0.06) | 0.00 (0.00, 0.44) | 0.3 | | |
| IPAQ (Mets) | 3655 (724, 6578) | 1548 (310, 3113) | 0.006 |  | 2057 (484, 4840) | 1740 (670, 4050) | 0.6 | |  | | -210 (-2183, 1286) | 475 (-488, 1256) | 0.11 | | |
| DASI (score) | 51 (31, 58) | 46 (35, 58) | >0.9 |  | 45 (28, 58) | 48 (29, 58) | 0.6 | |  | | 0 (-6, 0) | 0 (-8, 2) | 0.5 | | |
| DASI (Mets) | 8.97 (6.61, 9.89) | 8.42 (7.01, 9.89) | >0.9 |  | 8.23 (6.22, 9.89) | 8.61 (6.36, 9.89) | 0.5 | |  | | 0.00 (-0.73, 0.00) | 0.00 (-0.99, 0.28) | 0.5 | | |
| ^1^Median (interquartile range); ^2^Wilcoxon rank-sum test; **Abbreviations**: T4 = 12 months ; SF – PCS-12 = Short Form (Quality of life questionnaire) physical component score SF-PCS-12; SF – MCS-12 = Short Form (Quality of life questionnaire) mental component score – 12; MMAS-8 = Morisky Medication Adherence Scale in the 8-item version; IPAQ = Physical Activity Questionnaire Daily; DASI = Duke Activity Status Index. | | | | | | | | | | | | | |  |  |

## *Scenario 3: missing data from the control group were imputed using the most extreme negative values observed in the entire sample, while the most extreme positive values were used for the intervention group.*

### **Table SS23.** Change in clinical feature values between baseline and follow-up at 12 months.

|  | Intervention^1^  N = 44 | Control^1^  N = 50 | p-value^2^ |
| --- | --- | --- | --- |
| ∆Weight, Kg | -2.4 (-4.9, 0.4) | -3.0 (-4.7, -0.8) | 0.6 |
| ∆Cholesterol LDL, mg/dl | -17 (-48, -2) | -19 (-60, 8) | >0.9 |
| ∆Cholesterol non-HDL, mg/dl | -10 (-35, 4) | -10 (-28, 9) | 0.6 |
| ∆sBP, mmHg | -4 (-10, 7) | -5 (-15, 7) | 0.5 |
| ∆dBP, mmHg | 0 (-5, 6) | 0 (-8, 10) | >0.9 |
| ^1^Median (interquartile range); ^2^Wilcoxon rank-sum test; **Abbreviations**: ∆ = Difference between baseline and follow-up at 12 months;  Kg= kilograms; LDL = low-density lipoprotein; HDL = high-density lipoprotein; sBP = systolic blood pressure; dBP = diastolic blood pressure. | | | |

### **Table SS24.** Percentage of patients who achieved the therapeutic target for the parameters of interest.

|  |  | Baseline |  |  |  | T4 |  |
| --- | --- | --- | --- | --- | --- | --- | --- |
| Variables | Intervention  N = 44^1^ | Control  N = 50^1^ | p-value^2^ |  | Intervention  N = 44^1^ | Control  N = 50^1^ | p-value^2^ |
| HbA1c target | 0 (0%) | 0 (0%) | - |  | 22 (50%) | 21 (42%) | 0.4 |
| Hypoglicemic episodes | - | - | - |  | 1 (2.3%) | 4 (8.0%) | 0.4 |
| Lipid target | 22 (50%) | 22 (44%) | 0.6 |  | 33 (75%) | 29 (58%) | 0.08 |
| BP target | 32 (73%) | 37 (74%) | 0.9 |  | 35 (80%) | 36 (72%) | 0.4 |
| HbA1c +lipid+ BP combined target | - | - | - |  | 13 (30%) | 9 (18%) | 0.2 |
| ^1^number (%); ^2^Pearson's Chi-squared test; Fisher's exact test; **Abbreviations:** HbA1c = haemoglobin glycated; BP = blood pressure; T4 = 12 months. **Notes:** hypoglycemia is defined as a blood glucose level below 70 mg/dl or symptoms suggestive of it (e.g.shaking, sweating, dizziness); no baseline participants had a target HbA1c level (according to eligibility criteria). | | | | | | | |

### **Table SS25.** Baseline, 12-month, and changes (Δ) between the two time points in HbA1c levels

|  | **Baseline** | | |  | **T4** | | |  | | **∆(T4-Baseline)** | | | | |  |
| --- | --- | --- | --- | --- | --- | --- | --- | --- | --- | --- | --- | --- | --- | --- | --- |
| **Questionnaires** | **Intervention^1^** | **Control^1^** | **p-value^2^** |  | **Intervention^1^** | **Control^1^** | **p-value^2^** | |  | | **Intervention^1^** | **Control^1^** | **p-value^2^** | | |
|  | **N = 44** | **N = 50** |  |  | **N = 44** | **N = 50** |  | |  | | **N = 44** | **N = 50** |  | | |
| SF – PCS-12 | 50 (44, 54) | 51 (42, 55) | 0.7 |  | 53 (44, 54) | 49 (41, 54) | 0.2 | |  | | 1 (-1, 6) | 0 (-5, 3) | 0.2 | | |
| SF – MCS-12 | 54 (45, 58) | 51 (44, 56) | 0.084 |  | 55 (50, 59) | 51 (46, 56) | 0.027 | |  | | -3 (-7, 3) | -3 (-6, 5) | >0.9 | | |
| MMAS-8 | 8.00 (6.56, 8.00) | 7.38 (6.56, 8.00) | 0.3 |  | 8.00 (6.75, 8.00) | 8.00 (7.00, 8.00) | >0.9 | |  | | 0.00 (-0.06, 0.44) | 0.00 (0.00, 1.00) | 0.4 | | |
| IPAQ (Mets) | 3945 (973, 6797) | 1548 (310, 3113) | 0.002 |  | 2250 (838, 5820) | 1740 (670, 4050) | 0.2 | |  | | -143 (-2183, 1693) | 475 (-488, 1256) | 0.3 | | |
| DASI (score) | 51 (31, 58) | 46 (35, 58) | >0.9 |  | 45 (31, 58) | 48 (29, 58) | >0.9 | |  | | 0 (-5, 0) | 0 (-8, 2) | 0.9 | | |
| DASI (Mets) | 8.97 (6.61, 9.89) | 8.42 (7.01, 9.89) | >0.9 |  | 8.33 (6.59, 9.89) | 8.61 (6.36, 9.89) | >0.9 | |  | | 0.00 (-0.57, 0.00) | 0.00 (-0.99, 0.28) | 0.9 | | |
| ^1^Median (interquartile range); ^2^Wilcoxon rank-sum test; **Abbreviations**: T4 = 12 months ; SF – PCS-12 = Short Form (Quality of life questionnaire) physical component score SF-PCS-12; SF – MCS-12 = Short Form (Quality of life questionnaire) mental component score – 12; MMAS-8 = Morisky Medication Adherence Scale in the 8-item version; IPAQ = Physical Activity Questionnaire Daily; DASI = Duke Activity Status Index. | | | | | | | | | | | | | |  |  |

# **As-trated (AT) approach**

## *Scenario 0: missing data were imputed using the multiple imputation by chained equations (MICE) algorithm*

### **Table SS26.** Baseline, 12-month, and changes (Δ) between the two time points in HbA1c levels, by intervention and control groups.

|  | Baseline^1^ | T4^1^ | p-value^2^ | ∆ (T4- Baseline)^1^ | p-value^3^ |
| --- | --- | --- | --- | --- | --- |
| HbA1c, mmol/mol |  |  |  |  | 0.5 |
| Intervention | 59 (56, 66) | 54 (49, 58) | <0.001 | -6 (-11, -3) |  |
| Control | 63 (59, 71) | 54 (49, 63) | <0.001 | -7 (14, -2) |  |
| ^1^Median (interquartile range); ^2^Wilcoxon sign-ranked test HbA1c at baseline and at T4; ^3^Wilcoxon rank sum test comparing haemoglobin glycated changes between the two groups; **Abbreviations**: HbA1c = glycated haemoglobin. | | | | | |

### **Table SS27.** Change in clinical feature values between baseline and follow-up at 12 months.

| **Variables** | **Intervention^1^**  **N = 44** | **Control^1^**  **N = 59** | **p-value^2^** |
| --- | --- | --- | --- |
| ∆Weight, Kg | -2.4 (-4.9, 0.4) | -3.0 (-5.5, -0.9) | 0.5 |
| ∆Cholesterol LDL, mg/dl | -17 (-48, -2) | -27 (-63, 2) | 0.4 |
| ∆Cholesterol non-HDL, mg/dl | -10 (-35, 4) | -11 (-31, 5) | >0.9 |
| ∆sBP, mmHg | -4 (-10, 7) | -4 (-14, 9) | 0.6 |
| ∆dBP, mmHg | 0 (-5, 6) | 0 (-8, 7) | 0.8 |
| ^1^Median (interquartile range); ^2^Wilcoxon rank-sum test; **Abbreviations**: ∆ = Difference between baseline and follow-up at 12 months;  Kg= kilograms; LDL = low-density lipoprotein; HDL = high-density lipoprotein; sBP = systolic blood pressure; dBP = diastolic blood pressure. | | | |

### **Table SS28.** Percentage of patients who achieved the therapeutic target for the parameters of interest.

|  |  | **Baseline** |  |  |  | **T4** |  |
| --- | --- | --- | --- | --- | --- | --- | --- |
| **Variables** | **Intervention^1^**  **N = 44** | **Control^1^**  **N = 59** | **p-value^2^** |  | **Intervention^1^**  **N = 44** | **Control^1^**  **N = 59** | **p-value^2^** |
| HbA1c target | 0 (0%) | 0 (0%) | - |  | 22 (50%) | 26 (44%) | 0.6 |
| Hypoglicemic episodes | - | - | - |  | 1 (2.3%) | 4 (6.8%) | 0.4 |
| Lipid target | 22 (50%) | 26 (44%) | 0.6 |  | 33 (75%) | 36 (61%) | 0.14 |
| BP target | 32 (73%) | 41 (69%) | 0.7 |  | 35 (80%) | 41 (69%) | 0.3 |
| HbA1c +lipid+ BP combined target | - | - | - |  | 13 (30%) | 10 (17%) | 0.13 |
| ^1^number (%); ^2^Pearson's Chi-squared test; Fisher's exact test; **Abbreviations:** HbA1c = haemoglobin glycated; BP = blood pressure; T4 = 12 months. **Notes:** hypoglycemia is defined as a blood glucose level below 70 mg/dl or symptoms suggestive of it (e.g.shaking, sweating, dizziness); no baseline participants had a target HbA1c level (according to eligibility criteria). | | | | | | | |

### **Table SS29.** Baseline, 12-month, and changes (Δ) between the two time points in HbA1c levels.

|  | **Baseline** | | |  | **T4** | | |  | | **∆(T4-Baseline)** | | | | |  |
| --- | --- | --- | --- | --- | --- | --- | --- | --- | --- | --- | --- | --- | --- | --- | --- |
| **Questionnaires** | **Intervention^1^** | **Control^1^** | **p-value^2^** |  | **Intervention^1^** | **Control^1^** | **p-value^2^** | |  | | **Intervention^1^** | **Control^1^** | **p-value^2^** | | |
|  | **N = 44** | **N = 59** |  |  | **N = 44** | **N = 59** |  | |  | | **N = 44** | **N = 59** |  | | |
| SF – PCS-12 | 50 (44, 54) | 51 (42, 54) | 0.8 |  | 52 (41, 54) | 49 (43, 54) | 0.5 | |  | | 0 (-2, 6) | 1 (-5, 4) | 0.6 | | |
| SF – MCS-12 | 54 (45, 58) | 52 (45, 57) | 0.2 |  | 55 (50, 58) | 53 (46, 57) | 0.077 | |  | | -4 (-8, 3) | -4 (-8, 3) | >0.9 | | |
| MMAS-8 | 8.00 (6.56, 8.00) | 7.75 (6.63, 8.00) | 0.4 |  | 8.00 (6.75, 8.00) | 8.00 (6.75, 8.00) | >0.9 | |  | | 0.00 (-0.25, 0.44) | 0.00 (0.00, 0.38) | 0.4 | | |
| IPAQ (Mets) | 3655 (724, 6578) | 1980 (455, 3615) | 0.021 |  | 2147 (838, 5295) | 1305 (630, 3590) | 0.11 | |  | | -26 (-2078, 1478) | -5 (-1575, 1234) | 0.9 | | |
| DASI (score) | 51 (31, 58) | 45 (34, 58) | 0.9 |  | 45 (29, 58) | 47 (32, 58) | 0.6 | |  | | 0 (-5, 0) | 0 (-8, 5) | 0.5 | | |
| DASI (Mets) | 8.97 (6.61, 9.89) | 8.33 (6.96, 9.89) | >0.9 |  | 8.23 (6.27, 9.89) | 8.51 (6.66, 9.89) | 0.6 | |  | | 0.00 (-0.65, 0.00) | 0.00 (-0.98, 0.45) | 0.5 | | |
| ^1^Median (interquartile range); ^2^Wilcoxon rank-sum test; **Abbreviations**: T4 = 12 months ; SF – PCS-12 = Short Form (Quality of life questionnaire) physical component score SF-PCS-12; SF – MCS-12 = Short Form (Quality of life questionnaire) mental component score – 12; MMAS-8 = Morisky Medication Adherence Scale in the 8-item version; IPAQ = Physical Activity Questionnaire Daily; DASI = Duke Activity Status Index. | | | | | | | | | | | | | |  |  |

## *Scenario 1: no imputation was applied to the missing data*

### **Table SS30.** Baseline, 12-month, and changes (Δ) between the two time points in HbA1c levels, by intervention and control groups

|  | **Baseline^1^** | **T4^1^** | **p-value^2^** | **∆ (T4- Baseline)^1^** | **p-value^3^** |
| --- | --- | --- | --- | --- | --- |
| HbA1c, mmol/mol |  |  |  |  | 0.5 |
| Intervention | 59 (56, 66) | 54 (49, 58) | <0.001 | -6 (-11, -3) |  |
| Control | 63 (59, 71) | 54 (50, 63) | <0.001 | -7 (-14, -2) |  |
| ^1^Median (interquartile range); ^2^Wilcoxon sign-ranked test comparing HbA1c at baseline and at T4; ^3^Wilcoxon rank sum test comparing haemoglobin glycated changes between the two groups; **Abbreviations**: HbA1c = glycated haemoglobin. | | | | | |

### **Table SS31.** Change in clinical feature values between baseline and follow-up at 12 months

| **Variables** | **Intervention^1^**  **N = 44** | **Control^1^**  **N = 50** | **p-value^2^** |  |
| --- | --- | --- | --- | --- |
| ∆Weight, Kg | -2.1 (-4.9, 0.5) | -3.0 (-5.0, -1.0) | 0.4 |  |
| ∆Cholesterol LDL, mg/dl | -17 (-48, -2) | -24 (-67, 2) | 0.3 |  |
| ∆Cholesterol non-HDL, mg/dl | -10 (-35, 4) | -11 (-28, 2) | >0.9 |  |
| ∆sBP, mmHg | -4 (-10, 8) | -4 (-14, 7) | 0.4 |  |
| ∆dBP, mmHg | 0 (-5, 6) | 0 (-8, 7) | 0.8 |  |
| ^1^Median (interquartile range); ^2^Wilcoxon rank-sum test; **Abbreviations**: ∆ = Difference between baseline and follow-up at 12 months;  Kg= kilograms; LDL = low-density lipoprotein; HDL = high-density lipoprotein; sBP = systolic blood pressure; dBP = diastolic blood pressure. | | | | |

### **Table SS32.** Percentage of patients who achieved the therapeutic target for the parameters of interest.

|  |  | **Baseline** |  |  |  | **T4** |  |
| --- | --- | --- | --- | --- | --- | --- | --- |
| **Variables** | **Intervention**  **N = 44^1^** | **Control**  **N = 50^1^** | **p-value^2^** |  | **Intervention**  **N = 44^1^** | **Control**  **N = 50^1^** | **p-value^2^** |
| HbA1c target | 0 (0%) | 0 (0%) | - |  | 22 (50%) | 24 (43%) | 0.5 |
| Hypoglicemic episodes | - | - | - |  | 1 (2.3%) | 4 (6.8%) | 0.4 |
| Lipid target | 22 (50%) | 25 (43%) | 0.5 |  | 33 (75%) | 31 (56%) | 0.054 |
| BP target | 32 (73%) | 41 (69%) | 0.7 |  | 34 (79%) | 39 (71%) | 0.4 |
| HbA1c +lipid+ BP combined target | - | - | - |  | 13 (30%) | 9 (16%) | 0.12 |
| ^1^number (%); ^2^Pearson's Chi-squared test; Fisher's exact test; **Abbreviations:** HbA1c = haemoglobin glycated; BP = blood pressure; T4 = 12 months. **Notes:** hypoglycemia is defined as a blood glucose level below 70 mg/dl or symptoms suggestive of it (e.g.shaking, sweating, dizziness); no baseline participants had a target HbA1c level (according to eligibility criteria). | | | | | | | |

### **Table SS33.** Baseline, 12-month, and changes (Δ) between the two time points in HbA1c levels

|  | **Baseline** | | |  | **T4** | | |  | | **∆(T4-Baseline)** | | | | |  |
| --- | --- | --- | --- | --- | --- | --- | --- | --- | --- | --- | --- | --- | --- | --- | --- |
| **Questionnaires** | **Intervention^1^** | **Control^1^** | **p-value^2^** |  | **Intervention^1^** | **Control^1^** | **p-value^2^** | |  | | **Intervention^1^** | **Control^1^** | **p-value^2^** | | |
|  | **N = 44** | **N = 59** |  |  | **N = 44** | **N = 50** |  | |  | | **N = 44** | **N = 50** |  | | |
| SF – PCS-12 | 50 (44, 54) | 51 (42, 54) | 0.8 |  | 52 (42, 54) | 49 (43, 54) | 0.4 | |  | | 0 (-2, 5) | 0 (-5, 3) | 0.4 | | |
| SF – MCS-12 | 54 (45, 58) | 52 (45, 57) | 0.2 |  | 55 (49, 59) | 52 (46, 58) | 0.10 | |  | | -4 (-8, 2) | -4 (-6, 5) | 0.8 | | |
| MMAS-8 | 8.00 (6.56, 8.00) | 7.75 (6.63, 8.00) | 0.4 |  | 8.00 (6.75, 8.00) | 8.00 (7.00, 8.00) | 0.7 | |  | | 0.00 (-0.25, 0.25) | 0.00 (0.00, 0.50) | 0.2 | | |
| IPAQ (Mets) | 3810 (895, 6646) | 1980 (455, 3615) | 0.013 |  | 2147 (639, 5040) | 1283 (630, 3645) | 0.14 | |  | | -195 (-2100, 1440) | 23 (-1495, 1236) | 0.6 | | |
| DASI (score) | 51 (31, 58) | 45 (34, 58) | 0.9 |  | 45 (30, 58) | 46 (31, 58) | 0.8 | |  | | 0 (-5, 0) | 0 (-8, 5) | 0.4 | | |
| DASI (Mets) | 8.97 (6.61, 9.89) | 8.33 (6.96, 9.89) | >0.9 |  | 8.28 (6.38, 9.89) | 8.42 (6.61, 9.89) | 0.8 | |  | | 0.00 (-0.62, 0.00) | 0.00 (-0.98, 0.57) | 0.5 | | |
| ^1^Median (interquartile range); ^2^Wilcoxon rank-sum test; **Abbreviations**: T4 = 12 months ; SF – PCS-12 = Short Form (Quality of life questionnaire) physical component score SF-PCS-12; SF – MCS-12 = Short Form (Quality of life questionnaire) mental component score – 12; MMAS-8 = Morisky Medication Adherence Scale in the 8-item version; IPAQ = Physical Activity Questionnaire Daily; DASI = Duke Activity Status Index. | | | | | | | | | | | | | |  |  |

## *Scenario 2: missing data from the intervention group were imputed using the most extreme negative values observed in the entire sample, while the most extreme positive values were used for the control group*

### **Table SS34**. Baseline, 12-month, and changes (Δ) between the two time points in HbA1c levels, by intervention and control groups

|  | **Baseline^1^** | **T4^1^** | **p-value^2^** | **∆ (T4- Baseline)^1^** | **p-value^3^** |
| --- | --- | --- | --- | --- | --- |
| HbA1c, mmol/mol |  |  |  |  | 0.3 |
| Intervention | 59 (56, 66) | 54 (49, 58) | <0.001 | -6 (-11, -3) |  |
| Control | 63 (59, 71) | 54 (49, 62) | <0.001 | -8 (-16, -3) |  |
| ^1^Median (interquartile range); ^2^Wilcoxon sign-ranked test comparing HbA1c at baseline and at T4; ^3^Wilcoxon rank sum test comparing haemoglobin glycated changes between the two groups; **Abbreviations**: HbA1c = glycated haemoglobin. | | | | | |

### **Table SS35.** Change in clinical feature values between baseline and follow-up at 12 months

| **Variables** | **Intervention^1^**  **N = 44** | **Control^1^**  **N = 59** | **p-value^2^** |  |
| --- | --- | --- | --- | --- |
| ∆Weight, Kg | -2.1 (-4.8, 0.5) | -3.5 (-6.3, -1.0) | 0.11 |  |
| ∆Cholesterol LDL, mg/dl | -17 (-48, -2) | -31 (-83, 0) | 0.051 |  |
| ∆Cholesterol non-HDL, mg/dl | -10 (-35, 4) | -15 (-46, 1) | 0.3 |  |
| ∆sBP, mmHg | -3 (-10, 11) | -6 (-18, 6) | 0.13 |  |
| ∆dBP, mmHg | 0 (-5, 6) | 0 (-10, 6) | 0.3 |  |
| ^1^Median (interquartile range); ^2^Wilcoxon rank-sum test; **Abbreviations**: ∆ = Difference between baseline and follow-up at 12 months;  Kg= kilograms; LDL = low-density lipoprotein; HDL = high-density lipoprotein; sBP = systolic blood pressure; dBP = diastolic blood pressure. | | | | |

### **Table SS36.** Percentage of patients who achieved the therapeutic target for the parameters of interest.

|  |  | **Baseline** |  |  |  | **T4** |  |
| --- | --- | --- | --- | --- | --- | --- | --- |
| **Variables** | **Intervention**  **N = 44^1^** | **Control**  **N = 59^1^** | **p-value^2^** |  | **Intervention**  **N = 44^1^** | **Control**  **N = 59^1^** | **p-value^2^** |
| HbA1c target | 0 (0%) | 0 (0%) | - |  | 22 (50%) | 27 (46%) | 0.7 |
| Hypoglicemic episodes | - | - | - |  | 1 (2.3%) | 4 (6.8%) | 0.4 |
| Lipid target | 22 (50%) | 26 (44%) | 0.6 |  | 33 (75%) | 35 (59%) | 0.1 |
| BP target | 32 (73%) | 41 (69%) | 0.7 |  | 34 (77%) | 43 (73%) | 0.6 |
| HbA1c +lipid+ BP combined target | - | - | - |  | 13 (30%) | 13 (22%) | 0.4 |
| ^1^number (%); ^2^Pearson's Chi-squared test; Fisher's exact test; **Abbreviations:** HbA1c = haemoglobin glycated; BP = blood pressure; T4 = 12 months. **Notes:** hypoglycemia is defined as a blood glucose level below 70 mg/dl or symptoms suggestive of it (e.g.shaking, sweating, dizziness); no baseline participants had a target HbA1c level (according to eligibility criteria). | | | | | | | |

### **Table SS37.** Baseline, 12-month, and changes (Δ) between the two time points in HbA1c levels.

|  | **Baseline** | | |  | **T4** | | |  | | **∆(T4-Baseline)** | | | | |  |
| --- | --- | --- | --- | --- | --- | --- | --- | --- | --- | --- | --- | --- | --- | --- | --- |
| **Questionnaires** | **Intervention^1^** | **Control^1^** | **p-value^2^** |  | **Intervention^1^** | **Control^1^** | **p-value^2^** | |  | | **Intervention^1^** | **Control^1^** | **p-value^2^** | | |
|  | **N = 44** | **N = 59** |  |  | **N = 44** | **N = 59** |  | |  | | **N = 44** | **N = 59** |  | | |
| SF – PCS-12 | 50 (44, 54) | 51 (42, 54) | 0.8 |  | 51 (40, 54) | 49 (44, 55) | 0.9 | |  | | 0 (-2, 5) | 1 (-4, 4) | >0.9 | | |
| SF – MCS-12 | 54 (45, 58) | 52 (45, 57) | 0.2 |  | 54 (48, 58) | 53 (46, 58) | 0.6 | |  | | -4 (-8, 1) | -3 (-6, 5) | 0.5 | | |
| MMAS-8 | 8.00 (6.56, 8.00) | 7.75 (6.75, 8.00) | 0.5 |  | 7.75 (6.50, 8.00) | 8.00 (7.00, 8.00) | 0.2 | |  | | 0.00 (-0.31, 0.06) | 0.00 (0.00, 0.25) | 0.2 | | |
| IPAQ (Mets) | 3655 (724, 6578) | 1980 (455, 3615) | 0.024 |  | 2057 (484, 4840) | 1305 (630, 3740) | 0.4 | |  | | -210 (-2183, 1286) | 50 (-1487, 1241) | 0.4 | | |
| DASI (score) | 51 (31, 58) | 45 (34, 58) | 0.9 |  | 45 (28, 58) | 48 (32, 58) | 0.4 | |  | | 0 (-6, 0) | 0 (-7, 5) | 0.2 | | |
| DASI (Mets) | 8.97 (6.61, 9.89) | 8.33 (6.96, 9.89) | >0.9 |  | 8.23 (6.22, 9.89) | 8.70 (6.66, 9.89) | 0.4 | |  | | 0.00 (-0.73, 0.00) | 0.00 (-0.86, 0.60) | 0.2 | | |
| ^1^Median (interquartile range); ^2^Wilcoxon rank-sum test; **Abbreviations**: T4 = 12 months ; SF – PCS-12 = Short Form (Quality of life questionnaire) physical component score SF-PCS-12; SF – MCS-12 = Short Form (Quality of life questionnaire) mental component score – 12; MMAS-8 = Morisky Medication Adherence Scale in the 8-item version; IPAQ = Physical Activity Questionnaire Daily; DASI = Duke Activity Status Index. | | | | | | | | | | | | | |  |  |

## *Scenario 3: missing data from the control group were imputed using the most extreme negative values observed in the entire sample, while the most extreme positive values were used for the intervention group.*

### **Table SS38.** Baseline, 12-month, and changes (Δ) between the two time points in HbA1c levels, by intervention and control groups.

|  | Baseline^1^ | T4^1^ | p-value^2^ | ∆ (T4- Baseline)^1^ | p-value^3^ |
| --- | --- | --- | --- | --- | --- |
| HbA1c, mmol/mol |  |  |  |  | >0.9 |
| Intervention | 59 (56, 66) | 54 (49, 58) | <0.001 | -6 (-11, -3) |  |
| Control | 63 (59, 71) | 55 (50, 65) | <0.001 | -7 (-13, 0) |  |
| ^1^Median (interquartile range); ^2^Wilcoxon sign-ranked test comparing HbA1c at baseline and at T4; ^3^Wilcoxon rank sum test comparing haemoglobin glycated changes between the two groups; **Abbreviations**: HbA1c = glycated haemoglobin. | | | | | |

### **Table SS39.** Change in clinical feature values between baseline and follow-up at 12 months.

| **Variables** | **Intervention^1^**  **N = 44** | **Control^1^**  **N = 59** | **p-value^2^** |  |
| --- | --- | --- | --- | --- |
| ∆Weight, Kg | -2.4 (-4.9, 0.4) | -3.0 (-4.7, 0.0) | >0.9 |  |
| ∆Cholesterol LDL, mg/dl | -17 (-48, -2) | -19 (-63, 9) | >0.9 |  |
| ∆Cholesterol non-HDL, mg/dl | -10 (-35, 4) | -8 (-27, 10) | 0.3 |  |
| ∆sBP, mmHg | -4 (-10, 7) | -2 (-12, 12) | >0.9 |  |
| ∆dBP, mmHg | 0 (-5, 6) | 0 (-8, 10) | 0.6 |  |
| ^1^Median (interquartile range); ^2^Wilcoxon rank-sum test; **Abbreviations**: ∆ = Difference between baseline and follow-up at 12 months;  Kg= kilograms; LDL = low-density lipoprotein; HDL = high-density lipoprotein; sBP = systolic blood pressure; dBP = diastolic blood pressure. | | | | |

### **Table SS40.** Percentage of patients who achieved the therapeutic target for the parameters of interest.

|  |  | **Baseline** |  |  |  | **T4** |  |
| --- | --- | --- | --- | --- | --- | --- | --- |
| **Variables** | **Intervention**  **N = 44^1^** | **Control**  **N = 59^1^** | **p-value^2^** |  | **Intervention**  **N = 44^1^** | **Control**  **N = 59^1^** | **p-value^2^** |
| HbA1c target | 0 (0%) | 0 (0%) | - |  | 22 (50%) | 24 (41%) | 0.3 |
| Hypoglicemic episodes | - | - | - |  | 1 (2.3%) | 4 (6.8%) | 0.4 |
| Lipid target | 22 (50%) | 25 (42%) | 0.4 |  | 33 (75%) | 31 (53%) | **0.02** |
| BP target | 32 (73%) | 41 (69%) | 0.7 |  | 35 (80%) | 39 (66%) | 0.13 |
| HbA1c +lipid+ BP combined target | - | - | - |  | 13 (30%) | 9 (15%) | 0.08 |
| ^1^number (%); ^2^Pearson's Chi-squared test; Fisher's exact test; **Abbreviations:** HbA1c = haemoglobin glycated; BP = blood pressure; T4 = 12 months. **Notes:** hypoglycemia is defined as a blood glucose level below 70 mg/dl or symptoms suggestive of it (e.g.shaking, sweating, dizziness); no baseline participants had a target HbA1c level (according to eligibility criteria). | | | | | | | |

### **Table SS41.** Baseline, 12-month, and changes (Δ) between the two time points in HbA1c levels.

|  | **Baseline** | | |  | **T4** | | |  | | **∆(T4-Baseline)** | | | | |  |
| --- | --- | --- | --- | --- | --- | --- | --- | --- | --- | --- | --- | --- | --- | --- | --- |
| **Questionnaires** | **Intervention^1^** | **Control^1^** | **p-value^2^** |  | **Intervention^1^** | **Control^1^** | **p-value^2^** | |  | | **Intervention^1^** | **Control^1^** | **p-value^2^** | | |
|  | **N = 44** | **N = 59** |  |  | **N = 44** | **N = 59** |  | |  | | **N = 44** | **N = 59** |  | | |
| SF – PCS-12 | 50 (44, 54) | 51 (42, 54) | 0.8 |  | 53 (44, 54) | 49 (41, 53) | 0.09 | |  | | 1 (-1, 6) | 0 (-6, 3) | 0.09 | | |
| SF – MCS-12 | 54 (45, 58) | 52 (45, 57) | 0.2 |  | 55 (50, 59) | 51 (43, 57) | 0.014 | |  | | -3 (-7, 3) | -4 (-9, 3) | 0.5 | | |
| MMAS-8 | 8.00 (6.56, 8.00) | 7.00 (5.88, 8.00) | 0.14 |  | 8.00 (6.75, 8.00) | 7.75 (6.75, 8.00) | 0.6 | |  | | 0.00 (-0.06, 0.44) | 0.00 (0.00, 1.00) | 0.2 | | |
| IPAQ (Mets) | 3945 (973, 6797) | 1980 (455, 3615) | 0.008 |  | 2250 (838, 5820) | 1260 (615, 3590) | 0.053 | |  | | -143 (-2183, 1693) | -5 (1,575, 1234) | 0.8 | | |
| DASI (score) | 51 (31, 58) | 45 (34, 58) | 0.9 |  | 45 (31, 58) | 45 (28, 58) | 0.6 | |  | | 0 (-5, 0) | 0 (-9, 4) | >0.9 | | |
| DASI (Mets) | 8.97 (6.61, 9.89) | 8.33 (6.96, 9.89) | >0.9 |  | 8.33 (6.59, 9.89) | 8.23 (6.16, 9.89) | 0.6 | |  | | 0.00 (-0.57, 0.00) | 0.00 (-1.08, 0.45) | >0.9 | | |
| ^1^Median (interquartile range); ^2^Wilcoxon rank-sum test; **Abbreviations**: T4 = 12 months ; SF – PCS-12 = Short Form (Quality of life questionnaire) physical component score SF-PCS-12; SF – MCS-12 = Short Form (Quality of life questionnaire) mental component score – 12; MMAS-8 = Morisky Medication Adherence Scale in the 8-item version; IPAQ = Physical Activity Questionnaire Daily; DASI = Duke Activity Status Index. | | | | | | | | | | | | | |  |  |
